# The Genetic Information and Family Testing (GIFT) study: trial design and protocol

**DOI:** 10.1186/s12885-025-13744-6

**Published:** 2025-02-27

**Authors:** Steven J. Katz, Paul Abrahamse, Tim P. Hofer, Rebecca R. Courser, Rachel Hodan, Rachel S. Tocco, Sonia Rios-Ventura, Kevin C. Ward, Ann S. Hamilton, Allison W. Kurian, Lawrence C. An

**Affiliations:** 1 Department of Internal Medicine, University of Michigan, Ann Arbor, MI USA; 2 Department of Biostatistics, School of Public Health, University of Michigan, Ann Arbor, Mi, USA; 3 Department of Health Management and Policy, School of Public Health, University of Michigan, Ann Arbor, MI USA; 4 Department of Psychiatry, University of Michigan, Ann Arbor, MI USA; 5 Department of Medicine, Stanford University, Stanford, CA USA; 6 Department of Epidemiology and Population Health, Stanford University, Stanford, CA USA; 7 Cancer Genetics, Stanford Health Care, Stanford, CA USA; 8 Department of Epidemiology, Rollins School of Public Health, Emory University, Atlanta, Georgia; 9 Department of Preventive Medicine, University of Southern California, Los Angeles, USA

**Keywords:** Cascade, Genetic, Testing, Hereditary, Syndromes

## Abstract

**Background:**

There is pressing need to develop and evaluate clinically sound approaches to supporting the engagement between patients who have inherited cancer susceptibility and their relatives who may share it. Identifying and engaging patients with an inherited cancer susceptibility in the community is a potentially powerful strategy to reduce the gap in genetic risk evaluation for their families. The goal of the Genetic Information and Family Testing (GIFT) Study is to engage patients about inherited cancer susceptibility and provide support and services to their relatives to initiate genetic risk evaluation (including choice of home genetic testing).

**Methods/design:**

We are conducting a population-based, 2 × 2 factorial cluster-randomized clinical trial to implement and evaluate a direct-to-family, virtual, personalized, family-centered communication and decision-making tool: the Family Genetic Health Program. We use a unique SEER-based data infrastructure that we pioneered to identify patients diagnosed with cancer in the states of Georgia and California who carry a pathogenic variant (PV) in clinically tested cancer susceptibility gene. Eligible patients are offered enrollment into the trial and can invite their eligible first- and second-degree relatives to enroll. The index subject is randomized, and relatives are then cluster randomized by family. Participants in all arms receive some level of intervention, including at least the web-based platform with information about genetic testing and, for the relatives, an option to receive genetic testing through the study platform. We study the effects of two intervention design features: (1) the level of personalized family genetic risk navigation support: a technology-assisted, personally tailored patient and family member education and communication tool vs. the tool plus direct assistance from a lay human navigator); and (2) the cost of the genetic test offered to the relatives ($50 vs. free).

**Discussion:**

GIFT is a blueprint for how a virtual cascade genetic risk program can be delivered in the community, through a population-based approach to patients and relatives in families with hereditary cancer syndromes. The vision, experiences, and findings from GIFT will inform next-generation implementation science and the results will pertain to stakeholders interested in a population-based approach to cascade genetic risk evaluation.

**Trial registration:**

NCT05552664 registered at Clincaltrials.gov September 20, 2022.

**Supplementary Information:**

The online version contains supplementary material available at 10.1186/s12885-025-13744-6.

## Background

One of the most promising opportunities in cancer prevention today is to implement cascade genetic risk evaluation and management in families with an inherited susceptibility to cancer. There is growing evidence that implementing targeted, “cascade” genetic risk evaluation and management in families of patients with hereditary cancer susceptibility (HCS) may be the most cost-effective approach to reduce the population burden of cancer. Family risk is clustered around cancer cases with whom relatives share genes; the completeness and quality of cancer case ascertainment and reporting is very high in many regions of the United States (US); and detection of a pathogenic or likely pathogenic variant (PV) in a cancer susceptibility gene in a patient after diagnosis of cancer has high potential as an “intervenable moment” for engaging at-risk relatives through the process of cascade genetic risk evaluation (GRE). Thus, cancer case-based cascade GRE of relatives has emerged as the most promising approach to precision prevention and screening in the community, with growing endorsement by clinicians, specialty societies, and advocacy groups.

There is pressing need to develop and evaluate novel, clinically sound approaches to supporting the engagement between patients who have inherited cancer susceptibility and their family members who may share it. We among others have documented that the strong surge in multigene panel (MGP) testing after cancer diagnosis has fomented enormous challenges for patients, clinicians, and relatives – especially in families with HCS. At the same time, the clinical context of GRE after cancer diagnosis is increasingly complex. Guidelines today encompass many hereditary cancer syndromes, and more than one dozen cancer types based on growing evidence [1–6]. As MGP testing has become the norm, guideline organizations have converged on a list of > 40 cancer susceptibility genes in which PVs are clinically actionable, with wide variability in the threat and spectrum of cancer risk for relatives (e.g., recommended age of GRE), and options for prevention and screening (e.g., prophylactic surgery versus less invasive approaches).

A daunting challenge is that the patient with HCS is ultimately responsible to communicate with and engage their relatives in GRE. First- and second-degree relatives of a patient with a PV detected on genetic testing have a 50% and 25% probability, respectively, of carrying that PV; despite this shared health threat among at-risk relatives (ARRs), the social and contextual factors that affect family communication may vary enormously. Furthermore, ARRs are dispersed worldwide and embedded in disparate health care settings. Oncologists are necessarily focused on navigating treatment issues with patients after cancer diagnosis. Genetic Counselors are increasingly taxed and necessarily focused on engaging the many thousands of patients who have genetic testing annually. Indeed, there is a spirited debate about the need for formal pre-test genetic counseling given the paucity of Certified Genetic Counselors and the growing burden of post-test counseling, as PVs increasingly guide cancer treatment as well as prevention and screening. Furthermore, the patient’s insurance does not cover engagement of relatives in GRE. Taken together, there is no obligation, little incentive, and limited resources for clinicians to engage patients’ relatives. Given this lack of guidance, it is not surprising that most ARRs of cancer patients with PVs do not undergo clinically meaningful GRE [7–11].

Identifying and directly engaging patients with an inherited cancer susceptibility in the community is a potentially powerful strategy to reduce the gap in genetic risk evaluation in their families. The early survivorship period is an opportune time to implement communication and decision-making support between patients with HCS and their families because: (1) genetic testing in patients frequently occurs months after the initial diagnosis; (2) it is evident that large gaps in GRE in families persist; and (3) there is substantial interest in addressing cancer risk in the family as patients complete the arduous initial treatment course [12, 13].

We are conducting a population-based, pragmatic cluster-randomized clinical trial to implement and evaluate a direct-to-family, virtual, personalized, family-centered communication and decision-making tool - the Family Genetic Health Program (FGHP) - to close the gap in GRE and inform prevention and early detection strategies for relatives of patients with HCS. We use a unique Surveillance, Epidemiology, and End Results (SEER)-based data infrastructure that we pioneered to identify patients diagnosed with cancer in the states of Georgia and California who carry a PV in any clinically tested cancer susceptibility gene [14–16]. We target patients approximately three years after diagnosis in order to leverage our data infrastructure to: (1) identify a large, clinically and socially diverse cancer registry-based cohort of patients who tested positive for a clinically relevant germline PV; (2) use mature linked vital statistics data to identify patients alive ahead of our initial contact; and (3) use the Initiative data infrastructure to efficiently identify the gene(s) in which a PV is linked to the patient. We engage a clinically and socially diverse patient cohort with HCS and their family members using our virtual communication and decision-making approach, including an offer of clinical genetic testing and results reporting to first- and second-degree relatives, in partnership with a commercial laboratory (Color Health, Inc.) that has extensive experience with internet-based testing strategies in the US. This publication represents the documentation just prior the close of enrollment of the final protocol as carried out in the execution of the study. Where there are any important changes in execution of the protocol from the version submitted to our IRB dated 10/28/2022 (**see supplemental material**) we have noted them and discussed their potential impact in the text.

### Objectives

The goal of the GIFT Study is to support the capacity, opportunity, and motivation of cancer patients to engage their relatives about inherited cancer susceptibility; provide support and services to those relatives to initiate GRE (including choice of home genetic testing); and prepare relatives to engage their clinicians in informed decision-making about cancer prevention and early detection. GIFT features a web-based intervention that offers access to an online family communication program containing key facts about genetics, cancer risk, and the role of genetic testing. It also helps patients share health information with their first-and second-degree relatives, whom they can invite to join the study to receive education/support and access to low-cost genetic testing. Two design features of the intervention are randomized and evaluated to determine the best approach for future scalability. Those eligible are offered enrollment into the University of Michigan-hosted intervention trial, and those who enroll are randomized into one of four study arms (online platform for everyone; +/- human navigator; +/- $50 cost). Patients can invite their relatives to enroll and receive genetic testing via Color Health. Families randomized to the arms with human navigator support have access to a lay Family Health Navigator supported by our clinical cancer genetics research site based at Stanford University (AWK, RH). Enrolled patients and relatives are surveyed six months post-enrollment to collect additional information regarding their interactions with the GIFT platform and their experiences with genetic risk evaluation. The aims and hypotheses are:

### Primary Aim

**To determine the independent effects of the two virtual platform design features on relatives’ receipt of genetic testing.** We hypothesize that increased genetic risk navigation support and the availability of lower cost testing will increase the proportion of first- and second-degree relatives reported by patients on baseline survey who complete genetic testing through the study platform (primary outcome).

### Secondary aim 1

To determine the independent effects of the two virtual platform design features on the proportion of relatives invited by each patient to enroll in the study. We hypothesize that increased genetic risk navigation support and the availability of lower-cost testing will increase the proportion of first- and second-degree relatives reported by patients on a baseline survey who are invited by the patients to initiate GRE through the study platform (secondary outcome).

### Secondary aim 2

To determine the independent effects of the two virtual platform design features on cancer patients’ assessment of communication with their relatives about hereditary cancer and genetic risk evaluation. We hypothesize that increased genetic risk navigation support and the availability of lower-cost testing will substantially improve cancer patients’ assessment of their communication with relatives about hereditary cancer and genetic risk evaluation (secondary outcome).

### Secondary aim 3

To determine the independent effects of the two virtual platform design features on relatives’ receipt of a formal cancer genetic counseling session in practice. We hypothesize that increased genetic risk navigation support and the availability of lower-cost testing will increase the proportion of relatives enrolled in the trial that complete formal genetic risk evaluation with a clinician, after GIFT study participation.

### Study design

**Fig. 1:**
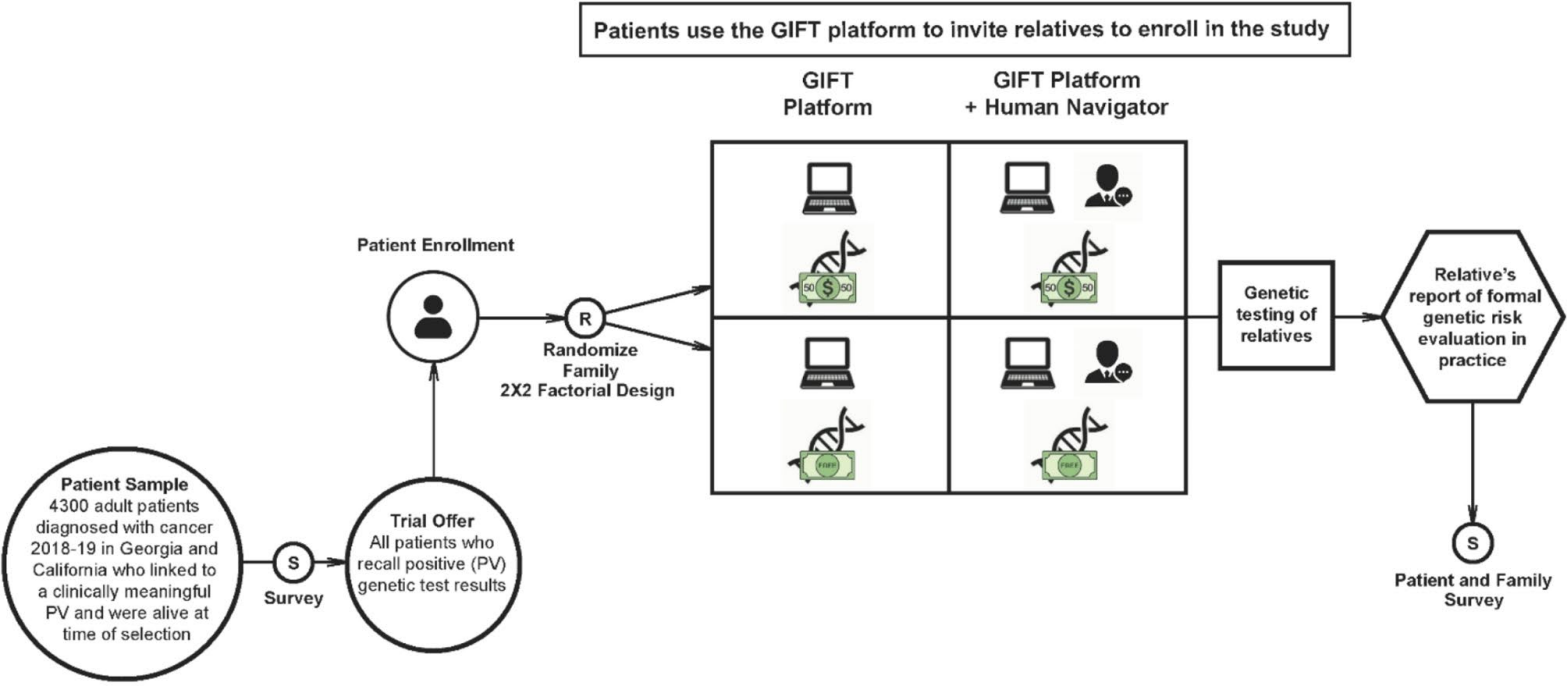
GIFT Study design and flow

The GIFT study begins with a patient inception cohort survey (PIC Survey) from which we determine eligibility for the clinical intervention trial. Eligible patients are offered enrollment into the trial and have the opportunity to invite their eligible first- and second-degree relatives to enroll. References throughout this paper are made to the “Patient Study” and the “Relatives Study” to more clearly describe the experiences of participants throughout this multilevel clinical trial. A follow-up survey is conducted six months post-enrollment for all enrolled patients and relatives, to collect data regarding intervention experiences and additional information needed to address secondary study outcomes. GIFT is a 2 × 2 factorial, prospective randomized clinical trial without a usual-care care arm (See Fig. 1 Study Design and Flow). The index subject (patient who has an eligible genetic PV) is randomized, and relatives are then considered cluster-randomized (by family), as it assures access to the same intervention arm (e.g., same test cost/access to Navigator) across all relatives in the index patient’s family. Participants in all arms receive some level of intervention, including at least the web-based platform with information about genetic testing and, for the relatives, an option to receive genetic testing through the study platform. We study the effects of two intervention design features: (1) the level of personalized family genetic risk navigation support: a technology-assisted, personally tailored patient and family member education and communication tool vs. the tool plus direct assistance from a human navigator); and (2) the cost of the genetic test offered to the relatives ($50 vs. free).

### Study patient population

Georgia and California SEER registry leads at Emory University and University of Southern California identify (via Information Management Services, as described below) an inception cohort of patients aged 18 and older diagnosed with a broad array of cancers in 2018–2019 who linked (via the ongoing Georgia California Genetic Testing Linkage Initiative) [14] to germline testing, and were found to have a pathogenic variant (PV) in one of 27 cancer susceptibility genes and were alive at the time of selection (*n* = 4300). Table 1 lists the genes included in the study, grouped by cancer susceptibility and guidelines for prevention and control at the time of selection of the patient sample [1].

**Table 1:** Pathogenic variants (PV) grouped by cancer susceptibility and guidelines for prevention and control at the time of selection of the patient sample

Cancer susceptibility	Current guidelines for prevention and control^2^	Genes (PV)
Breast cancer	Annual screening breast magnetic resonance imaging	*ATM,BRCA1,BRCA2,CDH1,CHEK2,PALB2,PTEN,STK11,TP53;* consider for *BARD1, BRIP1, RAD51C, RAD51D*
	Consider risk-reducing mastectomy	*BRCA1,BRCA2,PALB2,PTEN,STK11,TP53*
Colorectal cancer and/or other gastrointestinal cancers (e.g., gastric, esophageal junction)	Earlier and more frequent (every 1–5 years) colonoscopy and/or endoscopy	*APC, BMPR1A, CHEK2, EPCAM,GREM1, MLH1, MSH2, MSH6, POLD1, POLE, PTEN, SMAD4, STK11, TP53*
	Risk-reducing colectomy	*APC*
	Risk-reducing gastrectomy	*CDH1*
Ovarian and/or endometrial cancer	Risk-reducing salpingo-oophorectomy and/or hysterectomy	*BRCA1, BRCA2, BRIP1, EPCAM, MLH1, MSH2, MSH6, PTEN, RAD51C, RAD51D, STK11*
Prostate cancer	Earlier screening with annual PSA and clinical examination	*BRCA1, BRCA2;* consider for *ATM, CHEK2, PALB2*, and others
Pancreatic cancer	Consider annual screening protocols including endoscopic ultrasound and magnetic resonance cholangiopancreatography	*ATM, BRCA1, BRCA2, CDKN2A, EPCAM, MLH1, MSH2, MSH6, PALB2, STK11, TP53*
Melanoma/other skin cancer	Annual dermatologic examination	*BAP1, BRCA1/2, CDKN2A, CDK4, MITF, PALB2, PTEN, TP53, MLH1, MSH2, EPCAM, MSH6*
Other cancer sites (e.g., renal, thyroid, sarcoma)	Other targeted screening (e.g., thyroid ultrasound, renal ultrasound, whole body MRI)	*PTEN, TP53*, others

### Patient sampling

The SEER registries provide a third-party honest broker, Information Management Services, Inc. (IMS; the honest broker for the National Cancer Institute (NCI) Surveillance Program), with the de-identified clinical datasets maintained by the registries from the Georgia California Genetic Testing Linkage Initiative [14]. IMS samples an inception cohort of patients from this file using both clinical and genetic test result data according to the study eligibility criteria. IMS “salts” the sample with an additional 5% patients who did not link to a test result, which ensures that registry field teams do not know whether a given patient was tested or not, nor the result of any genetic test. After salting, IMS returns the patient IDs of the sampled individuals to the respective registry, with no information regarding genetic test status.

## Eligibility criteria

Inclusion criteria for the Patient Inception Cohort (PIC) survey are: (1) diagnosed with any cancer at any stage in 2018–2019 and reported to the Georgia or California SEER registries; (2) found to carry a pathogenic variant (PV) in one of 27 cancer susceptibility genes (see Table 1) according to the Georgia California Genetic Testing Linkage Initiative dataset; (3) aged 18 or older; (4) alive at the time of selection as determined through linkage with Georgia and California vital statistics data.

Additional eligibility criteria for Patient Trial Invitation are evaluated from patient response to the PIC survey and includes patients who report: (1) receipt of genetic testing for cancer risk and (2) a positive test result (pathogenic variant; PV).

Inclusion criteria for Relative Trial Invitation are assessed via patient report: (1) first-degree (biological parent, sibling, or biological child) or second-degree (biological half-sibling, aunt, uncle, nephew, niece, grandparent, or grandchild) relative of a patient enrolled in the study; (2) aged 18 or older; (3) alive at the time of study invitation; and (4) relative lives in the United States or Canada (countries in which Color Health genetic testing is available).

Additional eligibility criteria for Relative Trial Enrollment are evaluated from relative response to the relative eligibility screening survey and include: (1) confirmation from the relative that they have not received clinical genetic testing ordered by a doctor or genetic counselor within the past five years (proxy for having already been tested for the PV carried by the patient who invited them into the study); (2) confirmation of age 18 or older; (3) confirmation of first-degree (biological parent, sibling, or biological child) or second-degree (biological half-sibling, aunt, uncle, nephew, niece, grandparent, or grandchild) relation to the patient; and (4) confirmation of residence in United States or Canada.

**Fig. 2:**
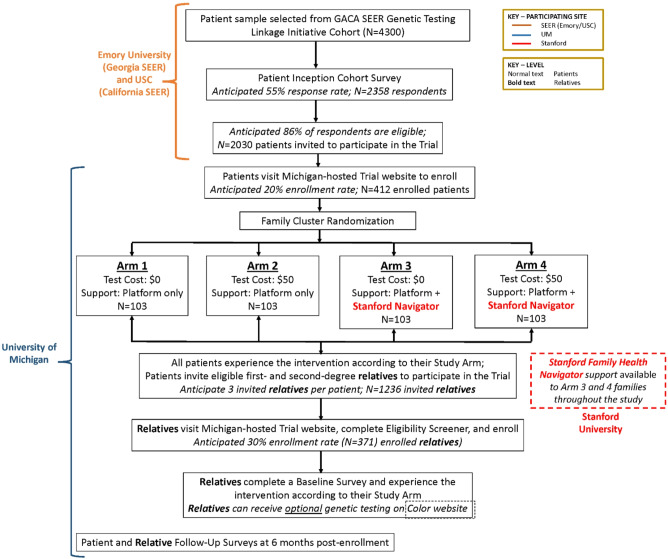
Summary of the GIFT study protocol

## Intervention

Randomization for this study occurs at the family cluster level – once a patient enrolls, they and their entire family are randomized to one of four study arms (See Fig. 2. Summary of the GIFT Study Protocol). The intervention experience is tailored to the family’s study arm in terms of the availability of the Stanford Family Health Navigator and the cost of genetic testing available through the platform to enrolled relatives.

## Patients

For the patient-level intervention, patients completing a survey and meeting eligibility criteria are given access to the online GIFT Study website, which is available to them for a total of six months. During that time, they can access it as many times, and for as much time, as they want. The website collects information from patients about their health and their family and is lightly tailored (personalized) based on information provided by the patients on the PIC survey (e.g., “You told us you have three sisters”). The intervention can pre-populate information reported by the patient on their PIC Survey because a third-party data scanning and entry service (DataForce, Inc.) is securely transferring completed survey data to Michigan for this purpose.

The study website guides patients through use of the intervention platform. Each feature is presented, and the patient is able to choose how deeply to engage with each one. Features available to patients in all four Study Arms include:

- Key facts and education about genetics, cancer risk, and the role of genetic counseling and testing for families with a history of cancer.
- The ability to invite family members via email to join the study (first 90 days only). Before each email invitation is sent, the patient has the ability to edit/personalize it. The patient is in control of what he/she shares via this invitation email to each relative and the only content that cannot be modified is the study description, which describes the study in terms of the family’s randomization assignment. To invite a relative via email, the patient enters their relative’s name/nickname, email address, and optional phone number into the study website and the email invitation is sent to the relative.
- A study “dashboard” with the ability to monitor their progress inviting relatives into the study. Patients may also be able to monitor which relatives have joined the study (if the enrolled relative gave us consent to share this back with the patient who invited them to the study). The dashboard also contains optional content around communication tips and strategies to follow-up with their relatives.

### Navigator services

Patients randomized to Study Arms 3 and 4 also have access the Stanford Family Health Navigator, via phone, email, and/or videoconference. The Navigator can help them use the website, answer questions, and invite family members to participate in the study. In addition to responding to requests for help from patients, the Navigator can also follow up with patients in Arms 3 and 4 proactively to encourage them to complete study tasks (e.g., invite more relatives). These communications listed here occur independently of the UM-hosted website.

## Relatives

For the relative-level intervention, relatives first complete a brief eligibility screener. Eligible relatives are guided through an electronic baseline survey to collect information needed to tailor their intervention experience and are then given access to the online GIFT Study website. It is available to them for a total of six months, and they can access it as many times, and for as much time, as they want.

The study website guides relatives through the use of the intervention platform. Each feature is presented and the relative is able to choose how deeply to engage with each one. Features available to relatives in all four Study Arms include:

Key facts and education about genetics and how it relates to cancer risk, the different types of genetic test results and their meaning, and privacy regarding genetic test results.

An exercise designed to help them consider their reasons for getting genetic testing (including commonly endorsed motivational statements and commonly endorsed hesitation statements). Motivational testimonials accompany this section to gently steer the reader in the direction of getting tested (which is clinically appropriate in this set of families with hereditary cancer syndromes).

A brief description of the Color Health genetic testing process and the ability to follow a unique link to order free ($0) or low-cost ($50) genetic testing from Color (according to their Study Arm) (first 90 days only).

The ability to consent to share the fact that they have enrolled in the study with the patient who invited them to join.

The study website contains a unique link to the Color website that embeds a unique Color alphanumeric token directly in the URL. This token allows Color to know how much to charge the relative participant for their genetic test ($50 vs. free) and that they are enrolled in the GIFT Study. No study participant PHI or PII is shared with Color using this token method. Relatives who click the link to order genetic testing follow Color’s standard procedures for ordering a genetic test, which includes informed consent for the testing procedure.

### Navigator services

Relatives randomized by family to Study Arms 3 and 4 have access to the Family Health Navigator, via phone/email/ videoconference, who can help them use the website, answer questions, and order genetic testing via Color. In addition to responding to requests for help from relatives, the Navigator can also follow up with relatives in Arms 3 and 4 proactively to encourage them to complete study tasks (e.g., complete their baseline survey; consider clicking the link to go to Color’s website to learn more about genetic testing).

## Recruitment and randomization

### Patient trial enrollment and family cluster randomization

Following invitation by the Georgia and California registries, interested patients can visit the University of Michigan-hosted study website to view and complete the guided web study sign-up process, which includes reviewing the online consent form and typing their name to electronically sign the informed consent document and creating an account on the website (to permit return at any time during the study window). Signature on the consent document indicates enrollment in the trial. We expect that approximately 20% of eligible and invited patients will enroll, for an expected total *n* = 412 patients enrolled in the randomized control trial (RCT). After enrollment, patients are randomized at the level of the family cluster (meaning that a patient and all of their relatives are randomized as a unit) into one of four trial arms that vary across two features of the intervention: (1) the level of personalized family genetic risk navigation support (online platform only vs. online platform + human navigator support) and (2) the cost of the genetic test option offered to relatives ($50 vs. free). Randomization is done using a computer program, with participants randomized to one of the four study groups using a random permuted-block design stratified by study site (Georgia and California).

Randomization is concealed from study participants; enrolled patients and relatives are not aware that their family has been randomized into one of four trial arms and provided a different intervention experience than the other study participants. Concealment is necessary for this study so that (a) the trial can observe differences across the trial arms without negatively impacting study enrollment and biasing the study, and (b) study participants do not experience unnecessary negative emotional reactions. The results of a pilot study have demonstrated that concealment can be effective – we did not observe a toxic or detrimental effect to enrollment for pilot study participants offered the $50 trial arm experience. There is no usual care arm in this study – all participants, regardless of randomization, receive the virtual intervention that includes education, assistance with family communication, and heavily discounted at-home testing for enrolled relatives.

### Relatives trial recruitment

Relatives are recruited to the study through the patient participants as part of the patient’s participation in the trial. Patients provide contact information for each first-degree (biological parent, sibling, or biological child) or second-degree (biological half-sibling, aunt, uncle, nephew, niece, grandparent, or grandchild) relative *whom the patient wishes to invite to the study*. As randomization for this study has already been conducted at the time of patient-level enrollment and is concealed from participants and potential participants as described earlier, methods of inviting relatives differ by study arm:

- **Patients in Arms 1 and 2** can invite relatives via email only.
- **Patients in Arms 3 and 4** can invite relatives via email and have the option to ask the Stanford Family Health Navigator to reach out by telephone and/or email to discuss the study with relatives.

*Email invitation*: Patients provide each relative’s name and email address and then review the draft email invitation. Portions of this invitation can be personalized (optional) by the patient for each relative prior to sending. Email invitations can be sent in both English and/or Spanish and are sent to the relative’s email address (as provided by the patient) via the trial platform. The email invitation includes: (1) a greeting from the patient inviting them to the study (customizable); (2) a brief description of the study; (3) a telephone number to call with questions or concerns; (4) a study website link for them to visit to learn more and enroll in the trial; and (5) their unique Access Code (study ID) (which can be linked back with the index patient in order to enable analysis of the entire family unit). A modified Dillman approach to encouraging trial enrollment is programmed into the study platform and sent via a series of email and/or text reminders to the relative at various timepoints.

#### Navigator invitation

Patients in Arms 3 and 4 can also ask the Family Health Navigator to help invite their relatives. Whether or not to involve the Navigator is on a per-relative basis. If the patient chooses Navigator invitation, they will also be asked to provide a phone number for that relative.

Relatives who have been invited with Navigator assistance receive email reminders encouraging enrollment as described above. In addition, the Navigator supplements these auto-emails with phone calls and/or personal emails to encourage enrollment. The Navigator routinely monitors the project dashboard and will not follow up with relatives who have already enrolled.

Regardless of study arm, all invited Relatives eventually receive an Invitation Email containing a clickable link to the GIFT Study website and the Relative’s unique Access Code. The study is described to relatives according to the family’s randomization assignment (study arm) and the fact of randomization assignment is concealed. Both patients and the Navigator can re-send email invitations as needed.

We expect that patient participants in the study (*N* = 412) will have on average a total of eight first-degree (biological parent, sibling, or biological child) and/or second-degree (biological aunt, uncle, nephew, niece, grandparent, or grandchild) adult relatives and that patients will be willing to provide contact information (email address) for three of those relatives (*N* = 1236).

### Relatives trial enrollment

Interested invited relatives visit the University of Michigan-hosted website for more information and complete a brief Eligibility Screener to confirm their eligibility for the study before entering the guided web study sign-up process, which includes typing their name to electronically sign the informed consent document and creating an account on the website (to permit return at any time during the study window). Signature on the consent document indicates enrollment in the study. Overall, we anticipate of those invited that 30% will enroll for a total *N* = 371. Relatives are unaware of their trial arm and experience an enrollment and the intervention consistent with the arm into which their family has been randomized.

## Summary sample size estimates

Table 2 summarizes the expected sample sizes for both Patients and Relatives in the GIFT Study revised prior to completion of patient enrollment (updated to clinicaltrials.gov 11/22/2024). Table 3 and 4summarizes the sample sizes for both Patients and Relatives that we expected prior to launch of the trial.

**Table 2:** Sample size summary estimates revised prior to completion of patient enrollment

Study phase	Sample Size
**Patients**	
Initial Patient Sample Selected	4,300 (approximate)
Respondents to PIC Survey	2,358 (expected response rate = 55%)
Pool of Patients eligible for GIFT Study Invitation	2,030 (86% of PIC survey respondents)
Patient GIFT Study Participants	412 (expected enrollment rate = 20%)
**Relatives**	
Relatives invited to the GIFT Study	1,236 (3.3 relatives per enrolled patient)
Relative GIFT Study Participants	371 (expected enrollment rate = 30%)

**Table 3:** Sample size summary estimates prior to launch of the trial

Study phase	Sample Size
**Patients**	
Initial Patient Sample Selected	5,250 (approximate)
Respondents to PIC Survey	3,150 (expected response rate = 60%)
Pool of Patients eligible for GIFT Study Invitation	2,930 (93% of PIC survey respondents)
Patient GIFT Study Participants	880 (expected enrollment rate = 30%)
**Relatives**	
Relatives invited to the GIFT Study	3,520 (4 relatives per enrolled patient)
Relative GIFT Study Participants	1,584 (expected enrollment rate = 30%)

### Measures

**Table 4:** Endpoints by study objectives

Objectives	Endpoints	Justification for Endpoint
**Primary Aims and Hypotheses**		
**Primary Aim**: To determine the independent effects of the two virtual platform design features on relatives’ receipt of genetic testing. We hypothesize that increased genetic risk navigation support and the availability of lower cost testing will increase the proportion of 1st and 2nd degree relatives reported by patients on baseline survey who complete genetic testing through the study platform (**primary outcome**).	**Family Genetic Testing Fraction**: The proportion of each enrolled patient’s first and second-degree relatives who receive Color genetic testing through the GIFT platform. For each enrolled patient, this will be calculated as the number of enrolled relatives who obtain a genetic test result from Color (complete the genetic testing process) via the GIFT Study divided by the number of relatives reported on the baseline PIC survey. The endpoint of interest is the presence (as opposed to the absence) of a test result (e.g., positive, uncertain, negative) on a quarterly report from Color Health. Assessed six months after the final relative enrolls in the study.	This is the most inclusive and pragmatic outcome relevant to cascade testing in practice and has high internal validity- that is, it is assessed uniformly across all trial arms. Providing six months for relatives to complete the Color testing process is pragmatic and based on observations made in our preliminary work with Color.
**Secondary Aims and Hypotheses**		
**Secondary Aim 1**: To determine the independent effects of the two virtual platform design features on the proportion of relatives invited by each patient to enroll in the study. We hypothesize that increased genetic risk navigation support and the availability of lower cost testing will increase the proportion of 1st and 2nd degree relatives reported by patients on a baseline survey who are invited by the patients to initiate GRE through the study platform (secondary outcome).	**Family Invite Fraction**: The proportion of each enrolled patient’s relatives who are invited to join the study. For each enrolled patient: Number of invited relatives / Number of relatives reported on baseline PIC survey Assessed 91 days after the final patient enrolls in the study.	This objective will illuminate how the trial features influenced patients’ willingness to invite eligible relatives. There will be complete ascertainment and uniform measurement across all trial arms. Assessment will occur on Day 91 after the final patient enrolls in the study because patients have 90 days to invite relatives.
**Secondary Aim 2**: To determine the independent effects of the two virtual platform design features on the cancer patients’ assessment of communication with their relatives about hereditary cancer and genetic risk evaluation. We hypothesize that increased genetic risk navigation support and the availability of lower cost testing will substantially improve cancer patient’s assessment of their communication with relatives about hereditary cancer and genetic risk evaluation (secondary outcome).	**Assessment of Family Communication Scale**: A 20-item scale with responses on a 5-point Likert from “not at all true” to “very true.” Items assess patients’ capacity, opportunity, and motivation to communicate with family members about their genetic test results. The outcome is continuous, and we will measure the change in mean score from baseline to follow-up survey. A greater difference between timepoints will indicate greater improvement in the patient’s assessment of their communication with relatives. Assessed at two time points: baseline PIC survey and Patient Six-Month Follow-up Survey.	This is an important patient-centered outcomes that will illuminate improvements to our next generation initiatives.
**Secondary Aim 3**: To determine the independent effects of the two virtual platform design features on relatives’ receipt of a formal cancer genetic counseling session in practice. We hypothesize that increased genetic risk navigation support and the availability of lower cost testing will increase the proportion of relatives enrolled in the trial that complete formal genetic risk evaluation in their medical practice within six months after enrollment.	**Relative Receipt of Formal Genetic Risk Evaluation** Single survey question (mark all that apply) with 3 potential responses. “Yes, Color” and “Yes, outside of Color” will indicate receipt of formal GRE. See Appendix F for details. Assessed in the Relative Six-Month Follow-Up Survey	This is an important relative-centered outcomes that will illuminate improvements to our next generation initiatives.
**Exploratory Aims**		
To explore the effect of the two virtual platform design features on the primary and secondary relatives testing outcomes described above across patient SES subgroups.	Family Genetic Testing Fraction and Family Invite Fraction – SES subgroup analyses. No new endpoints.	This is an important assessment of SES gradients in key outcomes across Trial arms that will inform whether there were disparities in the impact of the trial among patients and relatives.

**Other measures**: age, gender, education (from baseline cohort survey) census poverty level (from SEER)

### Data monitoring and collection

#### Patient and relative reported data

Data collected directly from patients and relatives (self-report survey data; patient-provided genetic testing results reports; relative contact information) is collected and stored online and initially managed by the University of Michigan Center for Health Communications Research (CHCR). The patient inception cohort have the option of completing the PIC survey via paper, which SEER study staff mail to a third-party data entry vendor (DataForce) for data entry. DataForce transfers the patient inception cohort survey data to CHCR via secure data transfer. The study website automatically records and saves relevant platform paradata (e.g., login, time spent per page, video watch counts). All personally identifiable information (PII) is stored in a separate file (linked to the participant’s unique Study ID), encrypted and password-protected, in order to manage study protocol adherence.

Study staff at the University of Michigan need access to the participant data stored by CHCR. All data is available via a password-protected project Dashboard Console built and administered by CHCR team members. Enrollment and study progress can be viewed directly on the console, and more detailed data files are available for download from the site as.csv files.

The Project Dashboard Console contains the following data elements and functionality:

- Enrollment flow.
- Protocol adherence (e.g., module completion; patient outreach to relatives; relative initiation of genetic testing).
- Survey status.
- Participant contact information (if outreach or additional reminders needed).

The study team may request data that goes beyond what is available on the Dashboard Console; any files generated in response to such a request is shared with the study team via Dropbox, a secure collaborative storage system that is approved for PHI by the University of Michigan Health System. The UM study team has access to project data through the Project Dashboard Console. The Data Manager works with Dr. An and the CHCR staff to maintain the highest quality datasets. All datasets include the participants’ unique Access Code (Study ID), allowing linkage of all datasets on an ongoing basis. The linking file is kept in an encrypted and password protected file, stored on a secure server at UM and accessible only to the study team.

#### Administrative, electronic and registry data

Other study data is obtained via linkage with external sources, including: (1) clinical, genetic test results, and demographic data from the Georgia California Genetic Testing Linkage Initiative (IMS), and (2) genetic testing information from Color Health for relatives who opt to receive genetic testing via the FGHP (limited to whether testing was processed and completed). Data use agreements, Health Insurance Portability and Accountability Act (HIPAA) authorizations, and Institutional Review Board (IRB) approvals are established as required to use these data for research purposes and participant consent is obtained before any linkage occurs. A third-party honest broker (IMS) links these datasets and create the analytic datasets. All data sets shared with CHCR contain only the unique Study IDs. Any other patient identifiers are not included.

All patient data, including survey, FGHP, and registry data, are combined using the unique Study IDs to create a patient level analytic dataset. Data for relatives is similarly combined to create a relative level analytic dataset. These datasets are stored and maintained by the project analytic and data management team on the University of Michigan enterprise cloud storage as the location for all databases. Analytic datasets are dynamically generated from raw de-identified data files using a shared code base that is curated by the study data management team and stored and updated centrally, ensuring that all analyses across projects are based on up-to-date data and coding conventions. Dropbox version control allows reinstating prior versions to ensure reproducibility of earlier results.

#### Data storage and management

All study data is collected and stored by CHCR at the University of Michigan, including the merged analytic datasets and their component data sources. CHCR maintains a philosophical and programmatic commitment to propagating the findings and products of our research. CHCR has an extensive history of supporting the sharing of research data from the perspective of fortifying the principles of scientific inquiry, facilitating alternative examinations of research questions, and avoiding duplicative data collection efforts. This study’s Leadership Team ensures that data sharing is undertaken with attention to human subjects protections, respect for proprietary data, and maintaining integrity of the source data.

To ensure that the data are protected, CHCR uses virtualized servers provided by Michigan Medicine’s Health Information Technology & Services (HITS). This Virtualization Service provides safer retention of data through storage and backup, physical security reducing the risk of damage or theft, and meets the regulatory requirements needed for this environment.

All servers and the back-end databases are password protected. The server runs the Red Hat Enterprise Linux operating system and security patches and updates are downloaded and installed regularly. Each server is also protected by firewalls to restrict network access to the server. When a participant accesses the study website, content is transmitted securely using secure socket layer (SSL) protocol, the same protocol used to protect financial and other personal information when transmitted from a web site to a user’s browser. This prevents anyone else on the network from intercepting and viewing the content that is being provided to the participant.

## External data sharing

This study randomizes family clusters into one of four trial arms. Two of those trial arms involve increased personalized support from a FGHP Navigator based at Stanford University. For those participants randomized to receive this support, UM share study data with Stanford University so that the Navigator can effectively support the family. Data are shared externally with Stanford University via the Project Dashboard Console described above. Participants consent to all external data sharing and only minimum necessary, de-identified datasets are shared.

## Data retention and destruction

The merged analytic datasets will stay live through the end of the grant period. All PII will be destroyed at the end of study period and only a de-identified set of crosswalk variables will be retained for purposes of linking the FGHP trial datasets to the IMS patient inception cohort data. At that time, all data stored in the database will be exported to.csv files and de-identified final datasets will be provided to the analytic team. No data will be used for the purposes of publication or public presentations without express permission of the Leadership Team.

## Quality assurance

The Data Manager assesses the PIC survey data (the only survey that is made available in paper format per the study protocol) for the degree of missing values on an ongoing basis during the initial patient survey fieldwork. Otherwise, we anticipate minimal missing data from that collected online, based on the prior expertise of CHCR in conducting and collecting online data.

## Participant safety monitoring

The study itself does not provide treatments of any kind. No products, medications or tests are being delivered as part of this study, though relatives are offered optional genetic testing services through the Program platform, and these are delivered by a third party, Color Health, for interested relatives. Identified potential risks to human subjects for this study include: (1) breach of confidentiality of PII and/or protected health information (PHI); (2) negative patient or relative reactions to study procedures; and (3) relatives’ difficulties dealing with the results of genetic testing received through Color Genomics as part of the FGHP.

The Leadership Team engages regularly with an appointed Data and Safety Monitoring Committee (DSMC) to monitor progress and benchmarks of the study. The DSMC is a multidisciplinary committee at the Rogel Cancer Center responsible for monitoring the safety and data integrity of appropriate cancer clinical research protocols conducted at the University of Michigan. The DSMC (1) reviews accrual information and determine whether and to whom outcome results should be released prior to the reporting of study results; (2) reviews reports of related studies to determine whether the monitored study needs to be changed or terminated; (3) reviews major proposed modifications to the study prior to their implementation (e.g., termination, dropping an arm based on reported trial outcomes, increasing target sample size); and (4) provides the Leadership Team with written information concerning findings for the trial as a whole related to cumulative adverse events observed and any relevant recommendations related to continuing, changing, or terminating the trial. A copy of this information will be provided to the NCI Division Director or designee. The Leadership Team provides information on cumulative adverse events and relevant recommendations to the local principal investigators to be shared with their IRBs.

## Adverse event management

We have been attentive to ensuring that there are processes in place to address any adverse events for participants in this study. Participation for both patients and relatives is completely voluntary, including the offer of genetic testing to relatives via Color Health. We collaborate closely with our partners at Stanford University and the SEER registries as well as the DSMC to proactively address any possible concerns. Any adverse events or unanticipated problems that do occur are discussed via the monthly Leadership Team meeting and/or the weekly team meetings. Participant confidentiality is always maintained. These events are recorded by the relevant site PI and reported to all IRBs of record and to the study sponsor. This is done by first submitting an “adverse event” amendment form to the UM IRB per protocol, followed by submission of similar forms to the other IRBs. Should any adverse events appear to be occurring frequently, we will amend the protocol to address these concerns. We do not anticipate having to stop the trial for these types of adverse events should they occur.

## Subject accrual and compliance

Review of the rate of subject accrual and compliance with inclusion/exclusion criteria occurs bi-weekly to monitor accrual progress. The DSMC reviews interim analyses of accrual and outcome data that are prepared by the analytic team and makes recommendations whether the study needs to be continued, changed, or terminated. In addition, the DSMC review and approve major modifications to studies that are proposed by the study team. All DSMC recommendations for study closure or study design changes are forwarded to the NCI for review and approval. In addition, the DSMC authorizes release of study data results. Progress reports, including patient and relative recruitment and retention/attrition are provided to the DSMC every three months. An annual quality assurance audit is conducted by the Quality Assurance Review Committee (QARC). QARC provides assurance that the trial is conducted, and data are collected, documented, and reported in compliance with the protocol and Good Clinical Practice Guidelines (GCP). The Annual Report is sent to the DSMC and is forwarded to the IRB and study sponsor. The IRB and other applicable recipients review progress of this study on an annual basis. The Contact PI (Katz) also sends copies of signed recommendations and comments from the DSMC to the sponsor Program Officer within one month of each monitoring review.

### Analysis plan

The analysis plan remains unchanged at the end of enrollment from that described in our IRB filing of 10/28/22 at the start of enrollment (see Study Protocol in Supplemental Material). As described above, we are testing the main effects of two interventions, represented by the separate platform design features of reduced vs. free test cost and web-based vs. human navigator communication and decision support. The 2 × 2 factorial design with block randomization at the level of the index patient (j) results in four groups of index patients of equal size. There are several analytic issues common to the primary and two secondary aims.

First, we do not hypothesize that there will be any synergistic effect of the two treatments on the log odds scale and thus assume that the effects will be additive. Conceptually we cannot think of any reason that the effect of the cost of the testing and any help provided by a navigator over the website have an obvious reason to be dependent on the level of the other. Thus, our primary outcome for the primary and secondary aims is the treatment effect of each intervention in a regression model without an interaction term.

The second analytic issue is that of informative cluster size. An assumption of the generalized linear multilevel models used in our analysis is that cluster size is not informative. The literature on family communication does not generally talk about family size as one of the factors that predicts health communication but there is little data one way or the other looking specifically at genetic testing. Thus, there is little to guide us on whether larger families are more or less likely to communicate well, or be likely to invite relatives, or whether relatives in larger families are more or less likely to get genetic testing when there is a potential health benefit. Thus, given the conditional independence assumption we will condition the models on cluster size with a threshold of including this covariate as a p value of 0.10 or less.

Finally, some of our analytic models will have other covariates including cluster size noted above and in some cases baseline levels of the outcome variable for models estimating change from a baseline variable. All of our models will include the stratification design variable for site (CA vs. GA). For models that include covariates we will estimate the marginal main effect of each treatment by averaging over the distribution of the covariates included in each model (such as baseline levels of outcome variables, state or cluster size). This in effect standardizes the treatment effect to a population with the distribution of those covariates observed in our study sample. For generalization purposes we have a fully population-based sample drawn from SEER registries. Thus, the baseline distribution of non-treatment covariates in our study sample is directly generalizable to the index patient population of the two large states from which the sample is drawn who are willing to enroll in a program to increase informed family participation in genetic testing. This is the target population of interest for any broader program that could be developed if the trial is successful.

Analysis plan for the Primary Aim (Family Genetic Testing Fraction), the dependent variable is the number of relatives who complete genetic testing from Color using a study-provided token. This outcome is the pre-specified primary outcome of the trial. We will describe a “*Family Genetic Testing Fraction*” as the proportion of eligible relatives of a patient who complete genetic testing. We anticipate that zero cost of the test and increased navigation support will be associated with a greater proportion of relatives completing testing, as quantified by the coefficients and standard errors of these covariates. For the Primary Aim, the target population is all eligible relatives who are identified by the index subject. The intervention is randomized by the index patient. We approach relatives only through the index patient, who will have an influence on the relatives’ responses based at a minimum on their prior relationship and most likely with direct communication about the study. Thus, the analysis for this Primary Aim will account for the cluster level randomization of the intervention and nesting of relatives within each index patient using multilevel generalized linear models. We will use a multilevel binomial regression model with a random intercept estimated for each index patient, the number of successes (relatives who are tested) as the outcome and the number of trials set as the number of eligible relatives. We will include the number of eligible relatives as a covariate in the model if the coefficient is significant (at the *p* = 0.10 level). We will also include relative sex and the patient-reported assessment of communication with family scale score to improve power as these baseline variables are likely to predict the outcome. No other variables will be included. The main effects of each intervention will be quantified by the marginal effect of the respective treatment variable averaging across the other treatment variable, covariates and cluster (eligible family) size.

For Secondary Aim 1 (Family Invite Fraction), the dependent variable is the number of a patient’s total eligible relatives for whom the patient is willing to provide contact information given the total number of eligible relatives (“*Family Invite Fraction*”). We expect that zero cost and increased navigation help will be associated with an increase in the proportion of relatives for whom a patient is willing to provide contact information, quantified by the coefficients and standard errors of these variables. We will use a multilevel binomial regression model with a random intercept estimated for each index patient, the number of successes (family members for whom an address is provided) as the outcome and the number of trials set as the number of eligible relatives. We will include the number of eligible relatives as a covariate in the model if the coefficient is significant (at the *p* = 0.10 level). We will also consider including the scale score of patient report of assessment of communication with family collected on the baseline PICS survey to improve power. No other variables will be included. The main effects of each intervention will be quantified by the marginal effect of each treatment variable averaging across the included covariates.

For Secondary Aim 2 (Assessment of Family Communication Scale), the target population of the intervention is the index cancer patient. The analytic sample includes all patients who consent and are randomized (expected *n* = 412). The dependent variable is the patient’s assessment of their communication with relatives (“*Patient assessment of family communication about genetic testing”)* at six-month follow-up, which will be measured as a continuous outcome using a multi-item scale. The treatment effect represents the change in assessment score from baseline to follow-up. We anticipate that increases in mean assessment score will be associated with zero cost and genetic risk navigation help. We will use linear regression to estimate the main effects of cost reduction and navigator help included as two treatment (1/0) variables. Each index patient’s baseline assessment score will be included as a covariate. We will include cluster size as a covariate if the coefficient is significant at the 0.10 level. The main effects of each intervention will be quantified by the marginal effect of each treatment variable averaging across the included covariates. The standard error of the main effect will define the confidence interval for the intervention effect.

### Sample size calculations

The number of enrolled subjects as achieved just prior to the close of enrollment was substantially lower than anticipated, however given considerable uncertainty in the planning stages about how successful our recruitment would be and the heterogeneity in the sample size requirements for the different aims, we had managed to design the trial such that the sample size for most of our aims were far larger than was needed to achieve a power of 0.8 with a alpha of 0.05.

Below we describe the calculations for power under the original and realized recruitment numbers.

For the primary aim we have two estimates, one for each of the two interventions in the factorial design.

### Assumptions

Effect sizes for the intervention were proposed to be the minimum clinically significant and achievable effect for assumed testing rates varying by trial features as shown in Table 5.

**Table 5:** Minimally detectable absolute difference between study arms

	No Navigator	Navigator
$50	10%	15%
Free	13%	18%

These probabilities reflect odds ratios of 1.4–1.5. To account for clustering in the relatives’ outcomes at the patient level, we assumed an estimate of 0.15 for the residual intra-class correlation (after accounting for the intervention effects).

**Fig. 3:**
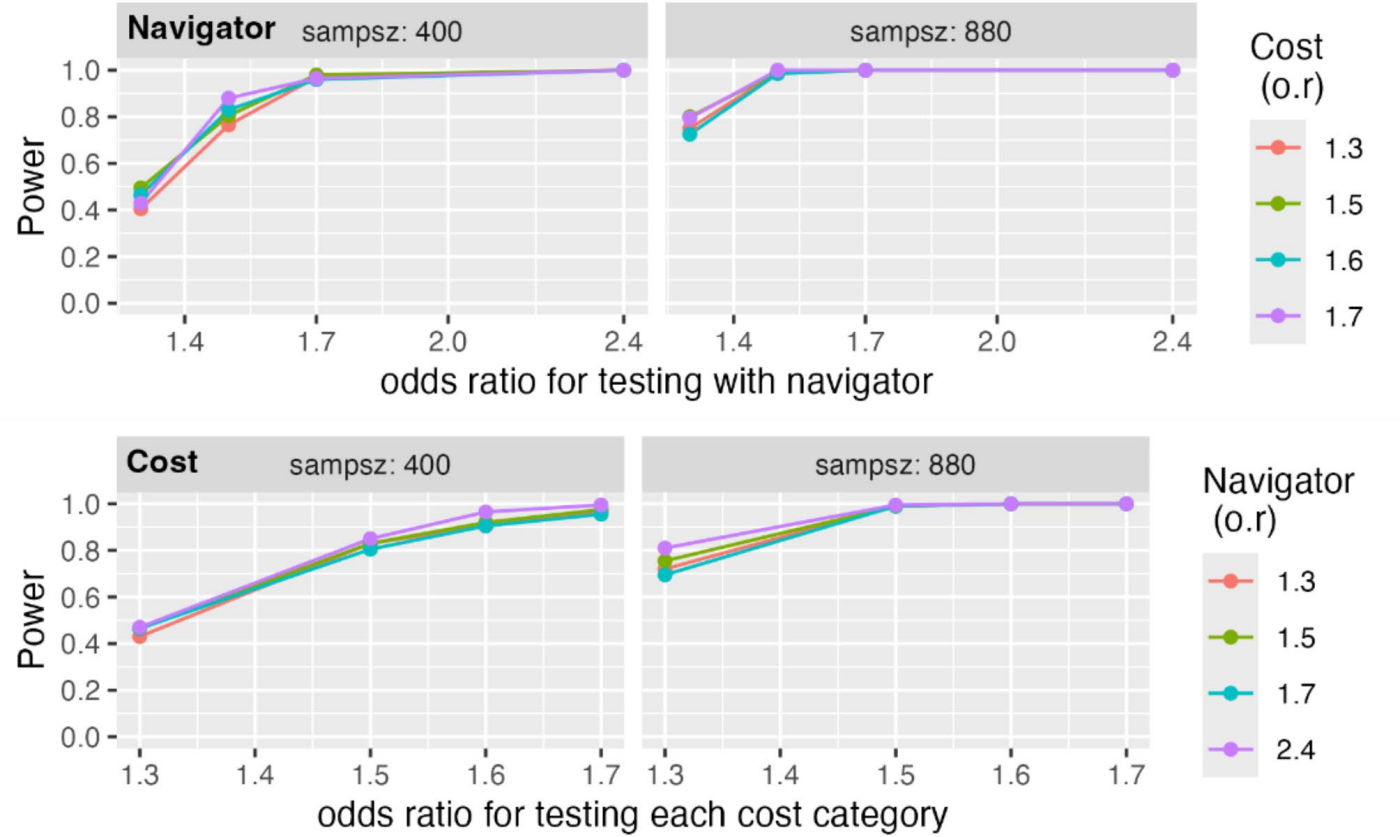
Power as a function of the odds ratio for the effect of low cost or navigator intervention at several levels of the effect of the second intervention

We originally hoped to enroll 880 index cancer patients with a median of 8 eligible relatives. As described in our original protocol, with these assumptions, a simulation based on our planned analytic approach confirmed a power of at least 0.80 for the effect of zero cost of the genetic test and navigation support for any effect including or above these specified effect sizes. However, we had built in a substantial buffer against our assumptions being different from what we proposed, including our enrollment numbers and the power was considerably higher than 0.80 for the larger sample size as seen in the graphs below and at an odds ratio of 1.5 we still have a power of 0.80 for the realized enrolled population of 400 or higher (Fig. 3).

These power calculations do not account for a feature of our design which will improve the efficiency of the estimation further, and that is the inclusion of a baseline measures that predict invitation and testing of family member as described above. The inclusion of such measures can substantially improve the efficiency of estimation of the intervention effect in any design. For cluster randomized trials such as this one, the effect of the baseline cluster level covariate on improving power increases as the ICC increases. Thus, at smaller ICCs than we assumed in our calculations above, our power will be substantially higher than shown. But if it is as high as assumed then the benefits from inclusion of the baseline covariate will be even larger [17].

The estimates will be more precise, and the power will be higher for secondary aim 1 (family invite fraction) as the proportion of relatives invited will be higher than those who get tested with the same denominator, as well as for the continuous outcome of secondary aim 2 (patient assessment of family communication) than for either of the binary outcomes.

### Descriptive aims

Unlike many randomized trials where a sample recruited at academic or other medical centers is not very generalizable, our sample is drawn with probability sampling from the entire population of patients tested for pathogenic variants in two large, diverse states. We have an opportunity to measure and describe a number of characteristics of the patient inception cohort using the SEER-based Georgia California Genetic Testing Linkage Initiative database which will be useful to examine the uptake of the intervention across different clinical and socioeconomic status (SES) subgroups (e.g., SEER Medicaid vs. other insurance, SEER census tract level SES, patient report of education level) and inform generalizability of our findings and the scaling of next-generation strategies.

## Discussion

The GIFT study is the first population-based randomized clinical trial to evaluate a direct-to-family, virtual, personalized, family-centered communication and decision-making tool. We have been highly successful at implementing all aspects of the study protocol as submitted at the time of initiation of the study including participant selection and enrollment procedures, the implementation of the intervention, and the collection of all pre-specified outcome measures. Our initial estimates of GIFT trial patient and relative participation documented in ClinicalTrials.gov prior to launch of the study were conservatively large, to ensure adequate power to detect even small differences across study arms, including potential interactions (see Table 3 above). Our revised estimates of GIFT trial enrollment described in the last amendment to clinicaltrials.gov prior to completion of the trial enrollment period (see Table 2 above) are sufficient to detect clinically meaningful difference across study arms. Lower than expected projections are due to several factors along the trajectory from patient selection through participant study flow. First, we sampled a lower number of patients than projected, largely due to overestimating the number of patients who were still alive at the time of selection (given a 2018–2019 diagnosis cohort) and some limitations on the project budget. Second, response rates to the baseline cohort survey were lower than expected, which has been a national trend in survey research during our study period largely due to barriers related to the peri-COVID-19 environment. Third, we overestimated the proportion of respondent patients who were eligible for the trial (who recalled a PV result on germline genetic testing). Fourth, we overestimated the expected proportion of eligible patients who would enroll in the GIFT trial (30% vs. 20%). The lower estimate appears to reflect patient uncertainty about the interest of family members in getting genetic testing, even though patients reported that very few relatives were tested at the time of completing their baseline survey. Finally, we also observed a lower-than-expected number of relatives invited by enrolled patients. Many enrolled patients reported feeling obligated to reach out to relatives before inviting them to GIFT, which influenced trial uptake in families. Enrolled patients’ uncertainty about providing relatives’ contact information was reduced over time through our persistent efforts to focus messaging on the value of the study for families, the quality of the team, and assurances of the protection of privacy. Invitation and enrollment of relatives is ongoing.

In summary, we view our experiences in this first large virtual intervention to deliver cascade cancer genetic risk education and testing in the community as a “glass half full”. Preliminary findings have shown that enrollees viewed their experiences favorably, but we did not achieve the participation we expected. The challenges we encountered are those that patients, families, and their clinicians face in practice every day. We have pinpointed the barriers to more robust uptake of the intervention, which will inform our next-generation intervention research: these include patients’ sense of the burden of obligation to contact relatives before being invited to our trial; patient reticence to re-visit prior informal and often clinically unsupported conversations with relatives; and the perception that the intervention has overemphasized an obligation to test vs. to deliver more effective cancer genetic risk education to relatives, so as to facilitate their shared decision-making about getting tested. However, as described in detail above, GIFT is sufficiently powered to detect clinically important differences across the study arms for the pre-specified primary and secondary outcomes. Our intervention experiences and outcomes inform strategies and tools to close the persistent gap in cascade genetic risk evaluation in families with hereditary cancer syndromes.

## Electronic supplementary material

Below is the link to the electronic supplementary material.

Supplementary Material 1

## Data Availability

No datasets were generated or analysed during the current study.

## Abbreviations

ARR: At-Risk Relatives
CHCR: Center for Health Communications Research
DSMC: Data and Safety Monitoring Committee
FGHP: Family Genetic Health Program
GCP: Good Clinical Practice Guidelines
GIFT: Genetic Information and Family Testing
GRE: Genetic Risk Evaluation
HBOC: Hereditary Breast and Ovarian Cancer
HCS: Hereditary Cancer Syndrome
HIPAA: Health Insurance Portability and Accountability Act
IMS: Information Management Services, Inc
IRB: Institutional Review Board
MGP: Multigene Panel
NCI: National Cancer Institute
PIC: Patient Inception Cohort
PV: Pathogenic Variant
QARC: Quality Assurance Review Committee
RCT: Randomized Control Trial
SEER: Surveillance, Epidemiology, and End Results
SES: Socioeconomic Status
SSL: Secure Socket Layer
UM: University of Michigan
US: United States
