# Supplementary material for: The Genetic Information and Family Testing (GIFT) study: trial design and protocol
Source: BMC Cancer. 2025 Feb 27;25:366. doi: 10.1186/s12885-025-13744-6 (PMC11869684; doi:10.1186/s12885-025-13744-6)
Supplement: Supplementary file 1 — Supplementary Material 1 [file 12885_2025_13744_MOESM1_ESM.pdf]

PROTOCOL UMCC 2022.066

The Genetic Information and Family Testing (GIFT) Study

Principal Investigator:

Steven Katz, MD (Contact PI)
Department of Internal Medicine
University of Michigan
2800 Plymouth Road
NCRC B16 Room 410E
Ann Arbor, MI 48109-2800
(Ph) 734-936-5292
skatz@med.umich.edu

Allison Kurian, MD, MSc
Department of Oncology
Stanford University
HRP Redwood Bldg Room T254A
150 Governor's Lane
Stanford, CA 94305-5405
(Ph) 650-724-7375
akurian@stanford.edu

Lawrence An, MD
Department of Internal Medicine
University of Michigan
2800 Plymouth Road
NCRC B16 Room G035E
Ann Arbor, MI 48109-2800
(Ph) 734-763-6099
lcan@med.umich.edu

Sub-Investigator(s):

University of Michigan

Sarah Hawley, PhD
Internal Medicine/General Medicine
University of Michigan

Lauren Wallner, PhD
Internal Medicine/General Medicine
University of Michigan

Tim Hofer, MD
Internal Medicine/General Medicine
University of Michigan

Stanford University

Rachel Hodan, MS, CGC
Department of Pediatrics
Stanford University

Emory University

Kevin Ward, PhD
Department of Epidemiology
Emory University

University of Southern California

Ann Hamilton, PhD
Population & Public Health Sciences
University of Southern California

Kathy Wojcik, PhD
Population & Public Health Sciences
University of Southern California

Biostatisticians:

Paul Abrahamse
Biostatistics, School of Public Health
University of Michigan

Allison Furgal
Biostatistics, School of Public Health
University of Michigan

Amended version: **10/28/2022**

Table of Contents

Study Schema 3
Detailed Study Schema 4
Study Summary 5
1.0 Objectives/Specific Aims 6
2.0 Background and Significance 8
3.0 Preliminary Data 11
4.0 Methods 12
4.1 Design 12
4.2 Subject Recruitment 13
4.3 Subject Selection 18
4.3.1 Inclusion Criteria 18
4.3.2 Exclusion criteria 19
4.4 Intervention 19
4.5 Time and Events Table 22
5.0 Measures 23
6.0 Procedures 25
6.1 Data Management 25
7.0 Data Analysis 28
7.1 Analysis Plan 28
7.2 Power Considerations 31
8.0 Protection of Human Subjects 31
9.0 Data and Safety Monitoring 34
10.0 Adverse Event Reporting 34
11.0 References 35
12.0 Appendices 37
A: Abbreviations 37
B. PICS Survey 38
C. Relative Eligibility Screener 50
D. Relative Baseline Measures 52
E. Patient Follow-Up Survey 53
F. Relative Follow-up Survey 56

STUDY SCHEMA

Figure 1. High-level GIFT Study Participant Flow, by Site*

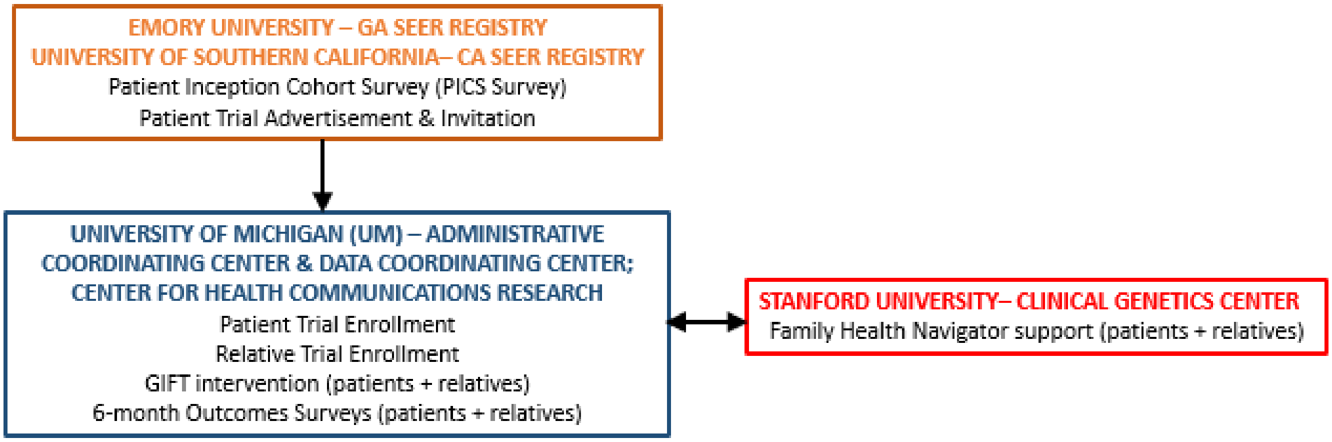

Figure 2. GIFT Study Detailed Schema*⁺

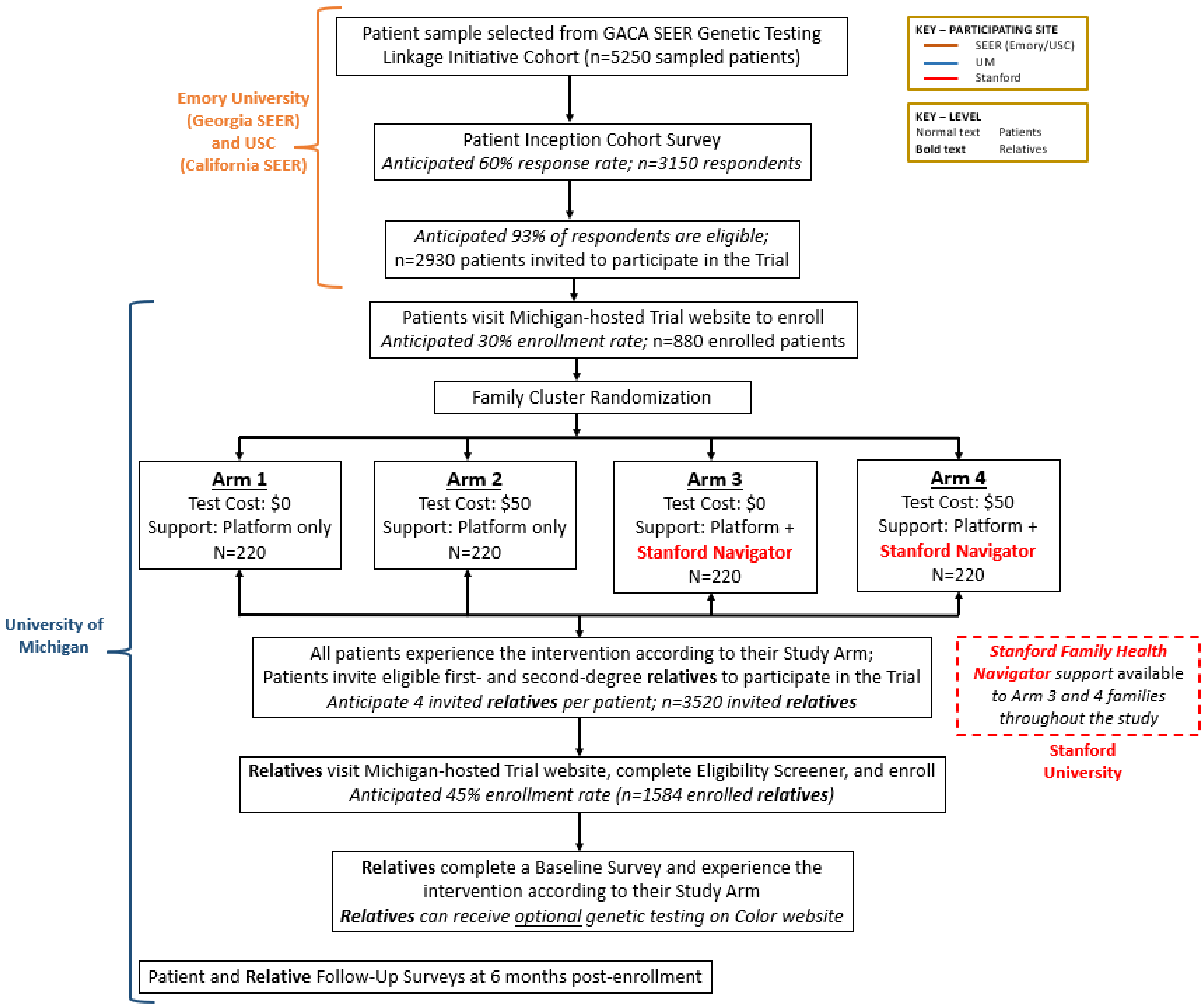

*No University of Michigan Rogel Cancer Center patients will be enrolled in this study. Project is not required to adhere to NIH sIRB policy and sites will obtain separate regulatory approval. An abbreviations key is provided as **Appendix A**.

⁺All patients who meet the study eligibility criteria and are alive at the time of sample selection will be selected into the study – the n=5250 sampled patients is an approximation. Vital status will be updated regularly and if the study learns a patient has died during the course of the study, they will not receive a survey.

STUDY SUMMARY

BACKGROUND AND SIGNIFICANCE: One of the most promising opportunities in cancer prevention is to implement cascade genetic risk evaluation (GRE) in families with hereditary cancer syndromes. There is growing evidence that targeting GRE in families where a pathogenic variant (PV) has been identified may be the most cost-effective approach to reduce the population burden of cancer through prevention. However, there are huge implementation challenges, such as: 1) The cancer patient is entirely responsible for communication and engagement of relatives for GRE; 2) relatives are dispersed worldwide and receive care in disparate health care practices; and 3) There is little incentive/limited resources for clinicians to engage with relatives and genetic counseling services are increasingly strained as the number of patients tested continues to grow. It is therefore not surprising that most relatives of cancer patients with PVs do not undergo GRE. The Genetic Information and Family Testing (GIFT) Study is designed to support the capacity, opportunity, and motivation of cancer patients to engage their relatives about inherited cancer susceptibility and provide support and services to those relatives to initiate GRE (including genetic testing) and prepare them to subsequently engage their clinicians in informed decision-making about cancer prevention and early detection.

INTERVENTION DESIGN: GIFT features a web-based intervention that offers access to an online family communication program containing key facts about genetics, cancer risk, and the role of genetic testing and helps patients share health information with their first-and second-degree relatives, whom they can invite to join the study to receive education/support and access to low-cost genetic testing. Two design features of the intervention will be randomized and evaluated to determine the best approach for future scalability: 1) the level of personalized family genetic risk navigation support (online program only vs. online program + human Navigator support) and 2) the cost of the genetic test offered to relatives ($50 vs free).

METHODS: GIFT is a 2x2 factorial RCT of a web-based intervention in a population-based sample of cancer patients and their relatives. We will identify and invite a SEER registry-based cohort of cancer patients with clinically relevant PVs and their families from Georgia (Emory University) and California (University of Southern California) to participate. **No University of Michigan Rogel Cancer Center patients will be enrolled in this study.** We will first perform a survey to determine study eligibility. Those eligible will be offered enrollment into the Michigan-hosted intervention trial, and those who enroll will be randomized (concealed; at the family cluster level) into 1 of 4 study arms. Patients can invite their relatives to enroll and receive optional $50 or free (according to the family's study arm) genetic testing via Color. Families randomized to the arms with human Navigator support will also have access to a Family Health Navigator at Stanford University. Enrolled patients and relatives will be surveyed six months post-enrollment to collect additional information regarding their interactions with the GIFT platform and their experiences with genetic risk evaluation. We will use platform paradata, SEER clinical and demographic data, Color genetic testing data, and patient and relative survey data to determine the independent effects of the two intervention design features on the primary outcome – the Family Genetic Testing Fraction – and on secondary outcomes that include the Family Invite Fraction, the Patient Assessment of Family Communication Scale, and the relatives' report of receipt of formal genetic risk evaluation post-intervention. Finally, we will explore the effect of the two virtual platform design features on the primary and secondary outcomes described above across patient SES subgroups.

IMPACT: The findings of this study have enormous potential to improve cancer prevention and early detection in families at high risk of hereditary cancer syndromes in the US.

1 Objectives/Specific Aims

One of the most promising opportunities in cancer prevention today is to implement cascade genetic risk evaluation in families with hereditary cancer syndromes. There is growing evidence that targeting genetic risk evaluation (GRE) in families where a cancer susceptibility gene (pathogenic variant (PV)) has been identified may be the most cost-effective approach to reduce the population burden of cancer through prevention. However, there are enormous challenges to implementing successful cascade GRE in families with hereditary cancer syndromes. We among others have shown that the strong surge in multigene panel (MGP) testing after cancer diagnosis has fomented enormous challenges for patients, clinicians and relatives. The clinical context of GRE after cancer diagnosis is increasingly complex: as MGP testing has become the norm, guideline organizations have converged on a list of more than 40 cancer susceptibility genes in which PVs are clinically actionable, with wide variability in cancer threat and a myriad of strategies for prevention and early detection. A daunting challenge is that the cancer patient is responsible for communication and engagement of relatives for GRE. Despite the shared health threat among at risk relatives (ARRs), the social and contextual factors that affect family communication are complex. Furthermore, ARRs are dispersed worldwide and receive care in disparate health care practices. Importantly, there is little incentive and limited resources for clinicians to engage cancer patients' relatives. And genetic counseling services are increasingly strained as the number of patients tested continue to grow. Given the lack of guidance for families, it is not surprising that most ARRs of cancer patients with PVs do not undergo GRE.

We will use a unique population-based data infrastructure we pioneered (the Georgia California Genetic Testing Linkage Initiative) to identify and invite a diverse cohort of cancer patients with clinically relevant PVs and their families to participate in our study. We will first perform a survey of approximately 5,250 cancer patients from our Georgia California Genetic Testing Linkage Initiative database: all patients (approximate n=5,000) who have a PV in one of 27 clinically relevant cancer susceptibility genes on genetic testing and an additional 5% random sample of patients who did not get tested (approximate N=250) in order to cloak the test status of the inception cohort to the SEER survey field teams. SEER field teams, blinded to the germline genetic testing status of the patient, will administer the patient inception cohort (PICS) survey that does not presume that patients have been tested or have a PV. The PICS Survey will serve as eligibility screener for the RCT.

Patients who are eligible upon survey will be invited to participate in our 2x2 RCT of a web-based intervention platform. As part of the study, enrolled patients will be able to invite their eligible first-and second-degree relatives to also enroll. Participants (at the family cluster level) will be randomized (in concealed fashion) into four groups and the study will evaluate the effects of two critical design features in order to determine the optimal approach for future scalability: 1) the level of personalized family genetic risk navigation support: a technology assisted, personally tailored patient and family member education and communication tool vs. the tool plus direct assistance from a human Navigator); and 2) the cost of the genetic test offered to the relatives ($50 vs free). Six months post-enrollment, enrolled patients and relatives will complete follow-up surveys to collection information regarding their experiences with the intervention and with formal genetic risk evaluation. **Table 1 describes the primary and secondary endpoints by study objectives.**

Table 1: Endpoints by Study Objectives		
**Objectives**	**Endpoints**	**Justification for Endpoint**
**Primary Aims and Hypotheses**		
**Primary Aim:** To determine the independent effects of the two virtual platform design features on relatives' receipt of genetic testing.<br><br>We hypothesize that increased genetic risk navigation support and the availability of lower cost testing will increase the proportion of 1st and 2nd degree relatives reported by patients on baseline survey who complete genetic testing through the study platform (**primary outcome**).	**Family Genetic Testing Fraction:** The proportion of each enrolled patient's 1st and 2nd degree relatives who receive Color genetic testing through the GIFT platform.<br>For each enrolled patient, this will be calculated as the number of enrolled relatives who obtain a genetic test result from Color (complete the genetic testing process) via the GIFT Study divided by the number of relatives reported on the baseline PICS survey. The endpoint of interest is the presence (as opposed to the absence) of a test result (e.g., positive, uncertain, negative) on the Color Quarterly Report.<br><br>Assessed six months after the final relative enrolls in the study.	This is the most inclusive and pragmatic outcome relevant to cascade testing in practice and has high internal validity- that is, it is assessed uniformly across all trial arms.<br><br>Providing six months for relatives to complete the Color testing process is pragmatic and based on observations made in our preliminary work with Color.
**Secondary Aims and Hypotheses**		
**Secondary Aim 1**: To determine the independent effects of the two virtual platform design features on the proportion of relatives invited by each patient to enroll in the study.<br><br>We hypothesize that increased genetic risk navigation support and the availability of lower cost testing will increase the proportion of 1st and 2nd degree relatives reported by patients on a baseline survey who are invited by the patients to initiate GRE through the study platform (secondary outcome).	**Family Invite Fraction:** The **p**roportion of each enrolled patient's relatives who are invited to join the study. For each enrolled patient:<br><br>Number of invited relatives / Number of relatives reported on baseline PICS survey<br><br>Assessed 91 days after the final patient enrolls in the study.	This objective will illuminate how the trial features influenced patients' willingness to invite eligible relatives. There will be complete ascertainment and uniform measurement across all trial arms. Assessment will occur on Day 91 after the final patient enrolls in the study because patients have 90 days to invite relatives.
**Secondary Aim 2:** To determine the independent effects of the two virtual platform design features on the cancer patients' assessment of communication with their relatives about hereditary cancer and genetic risk evaluation.<br><br>We hypothesize that increased genetic risk navigation support and the availability of lower cost testing will substantially improve cancer patient's assessment of their communication with relatives about hereditary cancer and genetic risk evaluation (secondary outcome).	**Assessment of Family Communication Scale:** A 20-item scale with responses on a 5-point Likert from "not at all true" to "very true." Items assess patients' capacity, opportunity, and motivation to communicate with family members about their genetic test results. The outcome is continuous and we will measure the change in mean score from baseline to follow-up survey. A greater difference between timepoints will indicate greater improvement in the patient's assessment of their communication with relatives.<br><br>Assessed at two time points: baseline PICS survey and Patient Six-Month Follow-up Survey.	This is an important patient-centered outcomes that will illuminate improvements to our next generation initiatives.

**Secondary Aim 3:** To determine the independent effects of the two virtual platform design features on relatives' receipt of a formal cancer genetic counseling session in practice.<br><br>We hypothesize that increased genetic risk navigation support and the availability of lower cost testing will increase the proportion of relatives enrolled in the trial that complete formal genetic risk evaluation in their medical practice within six months after enrollment.	**Relative Receipt of Formal Genetic Risk Evaluation**<br><br>Single question on a binary yes/no scale. Yes will indicate receipt of formal GRE.<br><br>Assessed in the Relative Six-Month Follow-Up Survey	This is an important relative-centered outcomes that will illuminate improvements to our next generation initiatives.
**Exploratory Aims**		
To explore the effect of the two virtual platform design features on the primary and secondary relatives testing outcomes described above across patient SES subgroups.	Family Genetic Testing Fraction and Family Invite Fraction– SES subgroup analyses. No new endpoints.	This is an important assessment of SES gradients in key outcomes across Trial arms that will inform whether there were disparities in the impact of the trial among patients and relatives.

Impact: The findings of this study have enormous potential to improve cancer prevention and early detection in families at high risk of hereditary cancer syndromes in the US and will be of great interest to a broad set of stakeholders including patients and their families, clinician groups, and population cancer registry leaders.

2 Background and Significance

One of the most promising opportunities in cancer prevention today is to implement cascade genetic risk evaluation and management in families with an inherited susceptibility to cancer. There is growing evidence that implementing targeted, "cascade" genetic risk evaluation and management (GRE) in families of patients with hereditary cancer susceptibility (HCS) may be the most cost-effective approach to reduce the population burden of cancer.[1,2] Family risk is clustered around cancer cases with whom relatives share genes; the completeness and quality of cancer case ascertainment and reporting is very high in many regions of the United States (US); and detection of a pathogenic variant (PV) in a cancer susceptibility gene in a patient after diagnosis of cancer has high potential as an "intervenable moment" for engaging at-risk relatives through the process of cascade GRE. While there has been limited endorsement of a BRCA1/2-only testing strategy in US[3] primary care, no guidelines endorse population screening using panels of multiple cancer susceptibility genes (multi-gene panels, MGP), which as we previously reported,[4] is the standard cancer genetic testing approach today. Thus, cancer case-based cascade GRE of relatives has emerged as the most promising approach to precision prevention and screening in the community, with growing endorsement by clinicians, specialty societies, and advocacy groups.

There is pressing need to develop and evaluate novel, clinically sound approaches to supporting the engagement between patients who have inherited cancer susceptibility and their family members who might. We among others

have documented that the strong surge in MGP testing after cancer diagnosis has fomented enormous challenges for patients, clinicians, and relatives – especially in families with HCS. We estimate that over 200,000 patients diagnosed with cancer this year in the US will undergo MGP testing and this number will continue to grow.[5] Nearly 15% (N=30,000) will have a clinically relevant PV that has important implications for their relatives. Thus, over the next decade, hundreds of thousands of cancer patients will be confronted with the need to engage relatives about HCS and the potential for GRE in their family to inform decisions about prevention and early detection. At the same time, the clinical context of GRE after cancer diagnosis is increasingly complex. Guidelines today encompass many hereditary cancer syndromes and more than one dozen cancer types. Indeed, scientific articles addressing the potential role of germline genetic testing in the management of different cancers appear weekly.[6-11] Furthermore, patients across a wide spectrum of disease severity must be engaged – from in situ breast cancer to advanced and metastatic cancers such as ovarian, lung, and pancreatic. As MGP testing has become the norm, guideline organizations[12-14] have converged on a list of >40 cancer susceptibility genes in which PVs are clinically actionable, with wide variability in the threat and spectrum of cancers (e.g., very high lifetime risk of multiple cancers with TP53 versus lower and more organ-specific risks with CHEK2), the impact of these risks for relatives (e.g., recommended age of GRE), and options for prevention and screening (e.g., prophylactic surgery versus less invasive approaches).

A daunting challenge is that the patient with HCS is ultimately responsible to communicate with and engage their relatives in GRE. First- and second-degree relatives of a patient with a PV detected on genetic testing have a 50% and 25% probability, respectively, of carrying that PV; despite this shared health threat among at-risk relatives (ARRs), the social and contextual factors that affect family communication may vary enormously. Furthermore, ARRs are dispersed worldwide and embedded in disparate health care settings. Oncologists are necessarily focused on navigating treatment issues with patients after cancer diagnosis. Genetic counseling is increasingly taxed and necessarily focused on engaging the many thousands of patients who have genetic testing annually. Indeed, there is a spirited debate about the need for formal pre-test genetic counseling given the paucity of genetic counselors and the growing burden of post-test counseling as PVs increasingly guide cancer treatment as well as prevention and screening. Furthermore, the patient's insurance does not cover engagement of relatives in GRE. Taken together, there is no obligation, little incentive, and limited resources for clinicians to engage patients' relatives. But where does this leave relatives who are confronted by the news of a loved one with cancer and now a PV conferring a cancer threat to the family? Do they engage their primary care doctors? Go online for direct-to-consumer testing such as 23andMe®? Do nothing? Given this lack of guidance, it is not surprising that most ARRs of cancer patients with PVs do not undergo clinically meaningful GRE.[15-17]

Identifying and directly engaging patients with an inherited cancer susceptibility in the community is a potentially powerful strategy to reduce the gap in genetic risk evaluation in their families. The early survivorship period is an opportune time to implement communication and decision-making support between patients with HCS and their families because 1) our own earlier work shows that genetic testing in patients frequently occurs months after the initial diagnosis 2) it is evident that large gaps in GRE in families persist; and 3) there is likely to be substantial interest in addressing cancer risk in the family as patients complete the arduous initial treatment course. We will target patients approximately two

years after diagnosis in order to leverage our unique Georgia California Genetic Testing Linkage Initiative data infrastructure to 1) identify a large, clinically and socially diverse cancer registry-based cohort of patients who tested positive for a clinically relevant germline PV; 2) use mature linked vital statistics data to identify patients alive at time of our initial engagement with them; and 3) use the Initiative data infrastructure to efficiently identify the gene in which a PV is reported by a patient. Given recent survival improvements from emerging targeted therapies[18] even for cancer types that previously had high one-year mortality (e.g., lung cancer, metastatic melanoma), this two-year time frame should enable us to capture a substantial majority of newly-diagnosed patients while they are still alive. Importantly, there is no deadline for GRE of ARRs, as the ultimate goal of GRE is precision screening and prevention over the life course.

Our study is very responsive to the recommendations from the Precision Prevention and Early Detection Working Group[19] and will accomplish key goals of the Beau Biden Cancer Moonshot NCI initiative, from which our team has obtained funding via a U01 award to conduct the current study. We will conduct a population-based, pragmatic cluster-randomized clinical trial to evaluate and disseminate a direct-to-family virtual solution: a personalized, family-centered communication and decision-making tool to close the gap in GRE and inform prevention and early detection strategies for relatives of patients with HCS. Our unique study is very timely, highly feasible, and has enormous potential to improve cancer prevention and early detection in relatives at high risk of HCS in the US. We can now use a unique SEER-based data infrastructure that we pioneered[4,20-23] to identify all patients diagnosed with cancer in the states of Georgia and California who carry a PV in any clinically-tested cancer susceptibility gene. We will engage a clinically and socially diverse patient cohort with HCS and their family members using a novel virtual communication and decision-making approach, including an offer of clinical genetic testing and results reporting to first- and second-degree relatives, in partnership with the commercial laboratory (Color Health, Inc.) that has the most extensive experience with internet-based testing strategies in the US. We have designed the study to maximize scalability with the potential to implement the strategies we develop across the entire SEER program over the next few years. Additionally, we anticipate that the virtual family communication and engagement intervention we develop will be attractive to clinical practices across the spectrum of specialties, including oncology, obstetrics/gynecology, gastroenterology, urology, and primary care – particularly those in underserved or rural areas with limited access to genetic counseling. The virtual platform we have developed will deploy tailored communication strategies to patients with PVs to support and facilitate their communication and engagement with their ARRs regarding HCS (including results disclosure) and to support ARRs' informed decision-making regarding completing formal GRE and potential prevention and early detection strategies. We will evaluate two key features of our intervention that will inform next-step scalability and implementation: 1) the value added by a human Family Health Navigator to facilitate communication between patients and ARRs and support ARRs' informed decision-making; and 2) the impact of different test costs offered to ARRs across the family clusters that reflect the range of charges for clinical testing by Clinical Laboratory Improvement Amendments (CLIA)-certified commercial test laboratories who are increasingly deploying direct-to-consumer strategies. We crafted real-world patient- and family-centered communication and decision-making outcomes highly responsive to the Cancer Moonshot initiative goals. Finally, our SEER-based sampling approach will ensure high representation of vulnerable populations such as racial and ethnic minority families and those living in low SES areas. Taken together, the results of this

study will be of enormous interest to many stakeholders, including the NCI Surveillance Program and regional SEER registry leaders, state health departments, clinicians, patient advocacy groups, and insurance plans.

3. **Preliminary Data**

Pilot Data: We conducted two separate pilot studies in preparation for conduct of the GIFT RCT. *HUM00189623* was a qualitative pilot study which involved interviews with genetic counselors as well as cancer survivors and their family members. In this pilot study, participants took part in a demonstration of a prototype GIFT intervention product. Participants were asked to “think aloud” as they moved through the demonstration and respond to interview questions and prompts from the study team. This pilot study collected feedback and usability information about the prototype intervention product to inform future development efforts and enrolled a total of eight participants from August – December 2021.

HUM00197640 was a SEER-based pilot study with our collaborators at Emory University. Participants were invited into the study using the intended study protocol and recruitment materials for the GIFT Study RCT. This pilot study assessed the overall feasibility of the study protocol – comparing patient and relative enrollment rates across study arms ($50 vs. free genetic test cost for relatives), collecting information about staff effort required to support the proposed trial protocol, and identifying any unanticipated challenges with the protocol. This pilot study enrolled 65 participants (36 patients and 29 relatives) from September 2021-May 2022. In response to lessons learned during the pilot study we have adjusted our anticipated trial enrollment rates, confirmed that a $50 genetic test cost is not detrimental to participant enrollment, and made significant improvements to the GIFT intervention product that we believe will maximize our likelihood of success. We also conducted qualitative debriefing interviews with pilot study participants to learn about their experience participating in this pilot study, which provided valuable input and testimonials as we designed the final intervention product and recruitment materials.

Study Team: Our highly experienced study team includes expertise in internal medicine, medical oncology and genetics, epidemiology, behavioral and implementation science, and biostatistics. We have a long collaboration history with more than 100 publications in cancer communication and decision-making research: many in high impact clinical journals. The University of Michigan (UM) will serve as the Administrative & Data Coordinating Center for the study, with collaboration from Stanford University, University of Southern California, and Emory University. **Table 2** shows key roles by institution.

Table 2. Project Personnel and Roles

**Name**	**Specialty**	**Project Role**
**University of Michigan – Administrative & Data Coordinating Center; Center for Health Communications Research (CHCR)**		
Steven Katz, MD, MPH	Medicine and health management	PI: Project implementation, survey design, data analysis, results interpretation, and dissemination
Lawrence An, MD	Medicine /CHCR Co-Director	PI: Project implementation, intervention design and development, and dissemination

Sarah Hawley, PhD, MPH	Health management /CHCR Co-Director	Co-I: Survey design and measures, intervention design, results interpretation, and dissemination
Lauren Wallner, PhD, MPH	Epidemiology and implementation science	Co-I: Intervention design, analysis planning, and dissemination
Tim Hofer, MD	Statistics and medicine	Co-I: Statistical design and methodology
Paul Abrahamse, MA	Biostatistics	Data Manager: dataset preparation, data analysis, results interpretation, and dissemination
Allison Furgal, MA	Biostatistics	Data Analyst: data analysis, results interpretation, and dissemination
**Stanford University – Clinical Genetics Center & Cancer Genetics Clinic**		
Allison Kurian, MD, MSc	Medical oncology and genetics	PI: Project implementation, oncology and clinical genetics expertise, and dissemination
Rachel Hodan, MS, CGC	Genetic counseling	Co-I: Oncology and clinical genetics expertise and dissemination
**University of Southern California (USC) – California SEER registry***		
Ann Hamilton, PhD	Epidemiology /California SEER registry	Co-I: Lead California SEER data, survey, and trial center activities and dissemination
Kathy Wojcik, PhD	Health behavior and epidemiology	Co-I: California SEER data, survey, and trial center activities and dissemination
**Emory University – Georgia SEER registry**		
Kevin Ward, PhD, MPH	Epidemiology /PI Georgia SEER	Co-I: Lead Georgia SEER data, survey, and trial center activities and dissemination

*Number of personnel in the California SEER registry reflects the higher workload, as the state comprises nearly 77% of patient inception cohort cases in this study.

4 Methods

4.1 Design

The GIFT study begins with a patient survey (PICS Survey) from which we will determine eligibility for the clinical intervention trial. Eligible patients will be offered enrollment into the trial and will have the opportunity to invite their eligible first- and second-degree relatives to also enroll. References throughout this protocol will be made to the Patient Study and the Relatives Study to more clearly describe the experiences of participants throughout this multilevel clinical trial. Six months-post enrollment for all enrolled patients and relatives, we will conduct follow-up surveys to collect data regarding intervention experiences and additional information needed to address secondary study outcomes. Please refer to Figures 1 and 2 for a visual illustration of the study structure and organization across sites.

The trial is a 2x2 factorial prospective RCT without a usual-care control arm. The index subject (patient who has an eligible genetic PV) is randomized, and relatives are then considered cluster randomized (by family), as they will receive the same intervention as the index subject who invited them. Participants in all arms receive some level of intervention including at least the web-based platform with information about genetic testing and, for the relatives, an option to receive genetic testing through the study platform (which would not be part of usual care unless an index subject sought out formal genetic counseling and was advised to encourage relatives get tested).

As shown in **Table 3** below, we will study the effects of two intervention design features: 1) the level of personalized family genetic risk navigation support: a technology assisted, personally tailored patient and family member education and communication tool vs. the tool plus direct assistance from a human Navigator); and 2) the cost of the genetic test offered to the relatives ($50 vs free).

Table 3: Study Arms

	Level of support	
Test Cost	Platform	Platform + Human Navigator
$0	**Arm 1**	**Arm 3**
$50	**Arm 2**	**Arm 4**

4.2 Subject Recruitment

Patient Sampling – occurs at Emory and USC

No University of Michigan Rogel Cancer Center patients will be enrolled in this study. Georgia and California SEER registry leads at Emory University and University of Southern California will identify (via IMS as described below) a **patient inception cohort of n=5,250 patients** aged 18 and older diagnosed with a broad array of cancers in 2018-2019 who linked (via the ongoing Georgia California Genetic Testing Linkage Initiative) to one of two testing statuses and were alive at the time of selection:

- 5,000 patients who were tested and found to have a pathogenic variant (PV) in one of 27 cancer susceptibility genes
  - 3,850 from California SEER
  - 1,150 from Georgia SEER
- 250 patients (5% of the n=5000 PV-positive patients in the first group) who were not tested (this is known as a 5% "salt"; done to cloak the testing status of patients from our SEER partners who will administer the survey)
  - 192 from California SEER
  - 58 from Georgia SEER

Note: This study will sample all eligible patients who, to the best of the study team's knowledge, are alive at the time of selection. The SEER sites may discover patients who have died during the course of the study – we will not send surveys to deceased patients. Thus, the sampling and surveying numbers provided in this protocol are the best possible approximation.

Table 4 lists the genes included in the study, grouped by cancer susceptibility and current guidelines for prevention and control.

**Table 4. Pathogenic variants (PV) grouped by cancer susceptibility and current guidelines for prevention and control**		
**Cancer Susceptibility**	**Current guidelines for prevention and control**[1,2,3]	**Genes (PV)**
**Breast cancer**	Annual screening breast magnetic resonance imaging	*ATM*, *BRCA1*, *BRCA2*, *CDH1*, *CHEK2*, *PALB2*, *PTEN*, *STK11*, *TP53;* consider for

		BARD1, BRIP1, RAD51C, RAD51D
	Consider risk-reducing mastectomy	*BRCA1*, *BRCA2*, *PALB2*, *PTEN*, *STK11*, *TP53*
**Colorectal cancer and/or other gastrointestinal cancers (e.g., gastric, esophageal junction)**	Earlier and more frequent (every 1-5 years) colonoscopy and/or endoscopy	*APC, BMPR1A, CHEK2, EPCAM*, *GREM1, MLH1, MSH2, MSH6, POLD1, POLE, PTEN, SMAD4, STK11, TP53*
	Risk-reducing colectomy	*APC*
	Risk-reducing gastrectomy	*CDH1*
**Ovarian and/or endometrial cancer**	Risk-reducing salpingo-oophorectomy and/or hysterectomy	*BRCA1, BRCA2, BRIP1, EPCAM, MLH1, MSH2, MSH6, PTEN, RAD51C, RAD51D, STK11*
**Prostate cancer**	Earlier screening with annual PSA and clinical examination	*BRCA1, BRCA2;* consider for *ATM, CHEK2, PALB2,* and others
**Pancreatic cancer**	Consider annual screening protocols including endoscopic ultrasound and magnetic resonance cholangiopancreatography	*ATM, BRCA1, BRCA2, CDKN2A, EPCAM, MLH1, MSH2, MSH6, PALB2, STK11, TP53*
**Melanoma/other skin cancer**	Annual dermatologic examination	*BAP1, BRCA1/2, CDKN2A, CDK4, MITF, PALB2, PTEN, TP53, MLH1, MSH2, EPCAM, MSH6*
**Other cancer sites** (e.g., renal, , thyroid, sarcoma)	Other targeted screening (e.g., thyroid ultrasound, renal ultrasound, whole body magnetic resonance imaging)	*PTEN, TP53,* others

The SEER registries will provide a third-party honest broker, Information Management Services, Inc. (IMS; the honest broker for the NCI Surveillance Program), with the de-identified clinical datasets maintained by the registries from the Georgia California Genetic Testing Linkage Initiative. IMS possesses the only crosswalk file that links these clinical datasets to the actual patient identifiers in the registries. IMS will sample an inception cohort of n=5000 patients from this file using both clinical and genetic test result data according to the study eligibility criteria. IMS will “salt” the sample with an additional 5% (n=250) patients who did not link to a test result, which will ensure that registry field teams will not know whether a given patient was tested or not, nor the result of any genetic test. After salting, IMS will return the patient IDs of the sampled individuals to the respective registry, with no information regarding genetic test status.

<u>Patient Recruitment – PICS survey – occurs at Emory and USC</u>

Each SEER site will verify vital status and current contact information for potential study participants and will ensure that all required IRB approvals are in place prior to patient contact. The SEER registries will then invite patients via postal mail to participate in the PICS survey, which will collect information required to determine trial eligibility and also, for patients who ultimately enroll, information needed to calculate primary and secondary outcomes (see **Appendix B**).

SEER staff will mail patients a packet of survey materials consisting of: 1) a letter written at the 7th-8th grade reading level indicating that they have been selected to participate in a study of cancer patients and describing its general purpose; 2) an information sheet with the elements of an informed consent; 3) a paper survey; 4) a $20 gift (cash or gift card); 5) a telephone number to call with questions or concerns; and 6) a postage-paid envelope for returning the survey. A duplicate copy of all survey packet materials will be provided in Spanish for all patients with suspected Hispanic ethnicity based on the NAACCR NHIA variable. If mail is returned as undeliverable, an attempt will be made to obtain an alternate mailing address and the survey packet will be sent to the updated address if one is found.

SEER sites will mail surveys in monthly batches and a modified version of the Dillman method will be used to encourage survey response:

- 2-3 weeks after the initial survey mailing, SEER site staff will begin calling individuals who have not completed the survey. The purpose of these calls and is to clarify questions, explain confidentiality procedures, and encourage participation. At least 5 calls will be made to reach the patient, including nights and weekends as necessary.
    - If patient is unable to be contacted due to unverifiable phone number, disconnected phone number, or no answer or voice mail indicating the correct number after multiple attempts, SEER study team will conduct additional tracing to try and find an updated mailing address and/or phone number.
- 2 weeks later, if patient still has not completed the survey and has not opted out, SEER study staff will send a reminder letter encouraging survey completion.
- For those who remain non-responders after 2 additional weeks, a second survey packet will be mailed (minus incentive).
- 2 weeks later, if still unsuccessful, SEER study team will make additional attempts to encourage survey completion via phone calls, texts, and reminder letters. This will continue to occur every 1-2 weeks until the 4-month mark.
    - Follow-up efforts will cease if a patient opts out or upon the passage of 4 months.
    - *Note:* Though follow-up efforts to increase survey response will cease after the 4-month mark for each batch, completed surveys received after this point in time will still be accepted until four months after the final batch of surveys is mailed.

As surveys are returned, SEER site staff will review for missing data. If items appear to be inadvertently missed (as opposed to intentionally skipped), staff will call back patients to obtain the missing information.

The total target N for PICS Survey completion is 60% or n=3,150 respondents.

<u>Patient Recruitment – 2x2 Trial</u>

Patient Trial Advertisement and Invitation (occurs at Emory and USC)

Patients will be deemed eligible for trial invitation if they self-report on the PICS Survey that they had germline genetic testing with a positive test result (indicating that they have a *PV conferring cancer susceptibility)*. **We expect approximately 93% of the PICS survey respondents to be considered eligible for trial invitation (n=2,930).**

SEER staff will access the project dashboard (see Section 6.1 Data Management for details) to learn which patients have been deemed eligible. They will then send eligible patients an invitation by postal mail and email (if known) to participate in the trial. The trial invitation offer will be written at the 7th-8th grade reading level, be provided in both English and Spanish (for those with suspected Hispanic ethnicity based on the NAACCR NHIA variable), and will 1) describe the study in general terms; 2) provide their unique Access Code (Study ID) and a telephone number to call with questions or concerns; and 3) instruct the patient to visit the GIFT study website in order to get additional information and to enroll. A paper study brochure that provides additional information about the study and study team will also be included in the mailing.

SEER teams will monitor the project dashboard to track which participants have enrolled and will conduct a modified version of the Dillman method (as described above) to encourage enrollment for those patients who have yet to do so. Efforts to convert enrollment will continue as described above until attempts to encourage enrollment have been exhausted (or until the patient enrolls or directly refuses).

Patient Trial Enrollment and Family Cluster Randomization (occurs at Michigan)

Interested patients will visit the University of Michigan-hosted study website to view and complete the guided web study sign-up process, which includes typing their name to electronically sign the informed consent document and creating an account on the website (to permit return at any time during the study window). Signature on the consent document indicates enrollment in the trial. **We expect that approximately 30% of invited patients will enroll, for an expected total n=880 patients enrolled in the RCT.** After enrollment, patients will be randomized at the level of the family cluster (meaning that a patient and all of their relatives will be randomized as a unit) into one of four trial arms that vary across two features of the intervention: 1) the level of personalized family genetic risk navigation support (online platform only vs. online platform + human Navigator support) and 2) the cost of the genetic test option offered to relatives ($50 vs free). Randomization will be done using a computer program, with participants randomized to 1 of the 4 study groups using a random permuted-block design stratified by study site (GA and CA). **See Table 5 below for projected enrollment by trial arm.**

Table 5. Projected Enrollment by trial arm – patient participants

	Level of support		
Test Cost	Platform	Platform + Human Navigator	TOTAL
$0	220	220	**440**
$50	220	220	**440**
	**440**	**440**	**880**

Randomization will be concealed from study participants; enrolled patients and relatives will not be aware that their family has been randomized into one of four trial arms and provided a different intervention experience than the other study participants. Concealment is necessary for this study so that the trial can observe differences across the trial arms (see Section 1 Study Objectives) without a) negatively impacting study enrollment and biasing the study, and b) causing unnecessary negative emotional reactions in study participants. The results of a pilot study (see Section 3 Preliminary Data) have demonstrated that concealment can be effective – we did not observe a toxic or detrimental effect to enrollment for pilot study participants offered the $50 trial arm experience. Importantly, there is no control arm or usual care arm in this study – ALL participants, regardless of randomization, receive the virtual intervention that includes education, assistance with family communication, and heavily discounted at-home testing for enrolled relatives. Study participants will not be debriefed after the completion of the study, as doing so would increase the risk of negative emotional responses to study procedures.

<u>Relatives Trial Recruitment – occurs at Michigan, with assistance from Stanford for Arms 3 & 4</u>

No University of Michigan Rogel Cancer Center patients will be recruited in this study. Relatives will be recruited to the study through the patient participants as part of the patient's participation in the trial. Patients will provide contact information for each first-degree (biological parent, sibling, or biological child) or second-degree (biological half-sibling, aunt, uncle, nephew, niece, grandparent, or grandchild) relative *whom the patient wishes to invite to the study*. As randomization for this study has already been conducted at the time of patient-level enrollment

and is concealed from participants and potential participants as described earlier, methods of inviting relatives differ by study arm:

- **Patients in Arms 1 and 2** will be able to invite relatives via email only.
- **Patients in Arms 3 and 4** will be able to invite relatives via email AND will also have the option to ask the Stanford Family Health Navigator to reach out by telephone and/or email to discuss the study and invite the relative to enroll.

Email invitation: Patients will provide each relative's name and email address and then review the draft email invitation. Portions of this invitation can be personalized (optional) by the patient for each relative prior to sending. Email invitations can be sent in both English and/or Spanish and will be sent to the relative's email address (as provided by the patient) via the trial platform. The email invitation includes: 1) a greeting from the patient inviting them to the study (customizable), 2) a brief description of the study, 3) a telephone number to call with questions or concerns; 4) a URL for them to visit to learn more and enroll in the trial; and 5) their unique Access Code (study ID) (which can be linked back with the proband patient in order to enable analysis of the entire family unit).

A modified Dillman approach to encouraging trial enrollment will be programmed into the study platform and sent via a series of email and/or text reminders to the relative at the following timepoints (if the relative enrolls, the email reminder schedule will be truncated):

- Days 3, 7, 14, 21, 30, 45, 60, 76, and 83 (opportunity for relatives to enroll ends at Day 90)

Navigator invitation: Patients in Arms 3 and 4 can also ask the Family Health Navigator to help invite their relatives. Whether or not to involve the Navigator is on a per-relative basis. If the patient chooses Navigator invitation, they will also be asked to provide a phone number for that relative.

Relatives who have been invited with Navigator assistance will undergo the same email reminder schedule as described above for email invitations. In addition, the Navigator will supplement these auto-emails with phone calls and/or personal emails to encourage enrollment. The Navigator will routinely monitor the project dashboard (see Section 6.1 for details) and will not follow up with relatives who have already enrolled.

The Navigator will encourage enrollment on or about the following schedule:

- Days 7, 30, and 60 (opportunity for relatives to enroll ends at Day 90)

Regardless of study arm, all invited Relatives will eventually receive an Invitation Email containing a clickable link to the GIFT Study website and the Relative's unique Access Code. The study is described to relatives according to the family's randomization assignment (study arm) and the fact of randomization assignment will be concealed. Both patients and the Navigator have the ability to re-send email invitations as needed.

We expect that patient participants in the study (n=880) will have on average a total of eight first-degree (biological parent, sibling, or biological child) and/or second-degree (biological aunt, uncle, nephew, niece, grandparent, or grandchild) adult relatives (n=7,040) and that patients will be willing to provide contact information (email address) for four of those relatives. Thus, **we anticipate that the UM-hosted trial platform will invite a total of 3,520 relatives to participate in the trial.**

<u>Relatives Trial Enrollment – occurs at Michigan</u>

Interested invited relatives will visit the Michigan-hosted website for more information and complete a brief Eligibility Screener (see **Appendix C**) to confirm their eligibility for the study before entering the guided web study sign-up process, which includes typing their name to

electronically sign the informed consent document and creating an account on the website (to permit return at any time during the study window). Signature on the consent document indicates enrollment in the study. Overall, we anticipate of those invited that 45% will enroll for a total N=1,584; **See Table 6 below.** Relatives will be unaware of their trial arm and will experience an enrollment and intervention experience consistent with the arm into which their family has been randomized.

Table 6. Projected enrollment by trial arm* – relative participants (n=1,584)

	Level of support		
Test Cost	Platform	Platform + Human Navigator	TOTAL
$0	327	401	**728**
$50	401	455	**856**
	**728**	**856**	**1,584**

*Table shows expected enrollment numbers per trial arm based on assumptions regarding the effect of each intervention arm on number of relatives invited by patients. Actual enrollment by arm will vary with sampling variability and is not controlled by the design or the study team.

<u>Summary</u>

Table 7 contains a summary of the expected sample sizes for both Patients and Relatives throughout the GIFT Study.

**Table 7. Sample Size Summary**	
**Study phase**	**Sample Size**
**Patients**	
Initial Patient Sample Selected	5,250 (approximate)
Respondents to PICS Survey	3,150 (expected response rate=60%)
Pool of Patients eligible for GIFT Study Invitation	2,930 (93% of PICS survey respondents)
Patient GIFT Study Participants	880 (expected enrollment rate=30%)
**Relatives**	
Relatives invited to the GIFT Study	3,520
Relative GIFT Study Participants	1,584 (expected enrollment rate=45%)

4.3 Subject Selection

4.3.1 Inclusion Criteria

<u>**Inclusion criteria for the PICS survey**</u> are:
1) diagnosed with any cancer at any stage in 2018-2019 and reported to the Georgia or California SEER registries

2) Been found to carry a pathogenic variant (PV) in one of 27 cancer susceptibility genes (see **Table 4**) according to the Georgia California Genetic Testing Linkage Initiative dataset

3) aged 18 or older

4) alive at the time of selection as determined through linkage with Georgia and California vital statistics data (to be updated routinely; surveys will not be sent knowingly to deceased patients)

Additional eligibility criteria for Patient Trial Invitation will be evaluated from patient response to the PICS survey and will include patient report of:

1) Receipt of genetic testing for cancer risk

2) A positive test result (pathogenic variant; PV)

Inclusion criteria for Relative Trial Invitation are assessed via patient report:

1) first-degree (biological parent, sibling, or biological child) or second-degree (biological half-sibling, aunt, uncle, nephew, niece, grandparent, or grandchild) relative of a patient enrolled in the study;

2) aged 18 or older;

3) alive at the time of study invitation;

4) relative lives in the United States, Canada, or Mexico (countries in which Color genetic testing is available, most people speak either English or Spanish, and test kit shipping costs are not exorbitant)

Additional eligibility criteria for Relative Trial Enrollment will be evaluated from relative response to the relative eligibility screening survey (see **Appendix C**) and will include:

1) confirmation from the relative that they have not received clinical genetic testing ordered by a doctor or genetic counselor within the past five years (proxy for having already been tested for the PV carried by the patient who invited them into the study);

2) confirmation of age 18 or older;

3) confirmation of first-degree (biological parent, sibling, or biological child) or second-degree (biological half-sibling, aunt, uncle, nephew, niece, grandparent, or grandchild) relation to the patient.

4) confirmation of residence in United States, Canada, or Mexico

4.3.2 Exclusion Criteria

Exclusion criteria for the PICS survey are:

1) Age<18

Additional exclusion criteria for Patient Trial Invitation:

1) Patients who do not report receipt of genetic testing

2) Patients who do not report a positive genetic test result (PV)

Exclusion criteria for Relative Trial Invitation are:

1) Age<18

2) Relative does not live in the United States, Canada, or Mexico

Additional exclusion criteria for Relative Trial Enrollment include relative report of:

1) Age<18;

2) Receipt of genetic testing ordered by a doctor or genetic counselor within the past five years;

3) Relationship to proband (inviting) patient other than first- or second-degree relative

4) Residence in a country other than United States, Canada, or Mexico

4.4 Intervention

Randomization for this study occurs at the family cluster level – once a patient enrolls, they and their entire family are randomized to one of four Study Arms (see **Table 3**). The intervention experience is tailored to the family's Study Arm in terms of the availability of the Stanford Family Health Navigator and the cost of genetic testing available through the platform to enrolled relatives.

Patients

For the patient-level intervention, patients will be given access to the online GIFT Study website. It will be available to them for a total of six months and they can access it as many times, and for as much time, as they want. The website will collect information from patients about their health and their family and will be lightly tailored (personalized) based on information provided by the patients on the PICS survey (e.g., "You told us you have three sisters"). The intervention is able to pre-populate information reported by the patient on their PICS Survey because a third-party data scanning and entry service (DataForce, Inc.) will be securely transferring completed survey data to Michigan for this purpose (see Section 6 Data Collection for more details).

The study website will guide patients through use of the intervention platform. Each feature will be presented and the patient will be able to choose how deeply to engage with each one. Features available to patients in all four Study Arms include:

- Key facts/education about genetics, cancer risk, and the role of genetic counseling and testing for families with a history of cancer
- The ability to invite eligible family members via email to join the study (**first 90 days only**). Before each email invitation is sent, the patient will have the ability to edit/personalize it. The patient is in control of what he/she shares via this invitation email to each relative and the only content that cannot be modified is the study description, which will describe the study in terms of the family's randomization assignment. To invite a relative via email, the patient will enter their relative's name/nickname, email address, and optional phone number into the study website and the email invitation will be sent to the relative.
- The ability to upload a copy/photo of their own genetic test results report/documentation of test results. If uploaded, this will influence their Relatives' experiences in the study (see "Relatives" section below; Relative intervention experience would be tailored to the specific PV indicated on their inviting patient's genetic test results report)

- A study “dashboard” with the ability to monitor their progress inviting relatives into the study. Patients may also be able to monitor which relatives have joined the study (if the enrolled relative gave us consent to share this back with the patient who invited them to the study). The dashboard will also contain optional content around communication tips and strategies to follow-up with their relatives.

Navigator services: Patients randomized to Study Arms 3 and 4 will also have the ability to speak with the Stanford Family Health Navigator, via phone, email, and/or videoconference. The Navigator can help them use the website, answer questions, and invite family members to participate in the study. In addition to responding to requests for help from patients, the Navigator will also follow up with patients in Arms 3 and 4 proactively to encourage them to complete study tasks (e.g., invite more relatives; upload a copy of their genetic test results report). These communications listed here occur independently of the UM-hosted website and will be reviewed and approved by Stanford University IRB prior to study initiation.

Relatives

For the relative-level intervention, relatives first complete a brief electronic baseline survey (see **Appendix D**) to collect information needed to tailor their intervention experience and are then given access to the online GIFT Study website. It will be available to them for a total of six months and they can access it as many times, and for as much time, as they want.

The study website will guide relatives through use of the intervention platform. Each feature will be presented and the relative will be able to choose how deeply to engage with each one. Features available to relatives in all four Study Arms include:

- Key facts/education about genetics, cancer risk, and the role of genetic counseling and testing for families with a history of cancer
    - If the relative’s inviting patient uploaded a copy of their genetic test results report, this section will be tailored to display information about the specific PV found in this relative’s family (primarily screening recommendations for individuals found to carry that PV).
    - If the proband did not upload a copy of their genetic test results, then this section will display information and screening recommendations for all genes included in this study and the relative can browse as deeply as they want.
- An exercise designed to help them consider their reasons for getting genetic testing (including commonly endorsed motivational statements and also commonly endorsed hesitation statements). Motivational testimonials will accompany this section to gently steer the reader in the direction of getting tested (which is clinically appropriate in this set of families with hereditary cancer syndromes)
- A brief description of the Color genetic testing process and the ability to follow a unique link in order to receive free ($0) or low-cost ($50) genetic testing from Color (according to their Study Arm) (**first 90 days only**)
- The ability to consent to share the fact that they have enrolled in the study back with the patient who invited them to join

- If the relative consents to share information about their study participation back with the patient who invited them to the study, that information will appear on the patient participant's GIFT Study website homepage. If the relative does not consent to share this information back with the patient who invited them, it will not be shared back.

The study website will contain a unique link to the Color website that will embed a unique Color alphanumeric token directly in the URL. This token will allow Color to know how much to charge the relative participant for their genetic test ($50 vs free) and that they are enrolled in the GIFT Study. No study participant PHI or PII is shared with Color using this token method. Relatives who click the link to order genetic testing will follow Color's standard procedures for ordering a genetic test, which includes informed consent for the testing procedure.

Navigator services: Patients randomized to Study Arms 3 and 4 will have the ability to speak with the Family Health Navigator, via phone/email/ videoconference, who can help them use the website, answer questions, and order genetic testing via Color Health. In addition to responding to requests for help from relatives, the Navigator will also follow up with relatives in Arms 3 and 4 proactively to encourage them to complete study tasks (e.g., complete their baseline survey; consider clicking the link to go to Color's website to learn more about genetic testing). These communications listed here occur independently of the UM-hosted website and will be reviewed and approved by Stanford University IRB prior to study initiation.

4.5 Time and Events Table

Table 8: Patients Time and Events Table

	Survey period Pre-intervention trial	Intervention period Months 1-3	Intervention period Months 4-6	Follow-up 6 months post-enrollment
PICS survey	X			
Trial eligibility determination	X			
Trial invitation	X			
Informed consent (Trial enrollment)	X			
Randomization (family cluster level)		X		
Patients can invite relatives		X		
Patient access to intervention		X	X	
Patient follow-up survey				X
"Behind the Scenes" data collection (no participant contact necessary)				
SEER clinical and demographic data	X			
Platform data (paradata)		X	X	X

Table 9: Relatives Time and Events Table

	Pre-study	Intervention period Months 1-3	Intervention period Months 4-6	Follow-up

				6 months post-enrollment
Eligibility survey	X			
Informed consent (Trial enrollment)	X			
Relatives baseline survey		X		
Relatives can order Color genetic testing		X		
Relatives' access to intervention		X	X	
Relatives follow-up survey				X
"Behind the Scenes" data collection (no participant contact necessary)				
Platform data (paradata)		X	X	X
Color quarterly report		X	X	X

5 Measures

Primary Outcome

Family Genetic Testing Fraction: The proportion of each enrolled patient's 1st and 2nd degree relatives who receive Color genetic testing through the GIFT study website. For each enrolled patient, this will be calculated in two steps:

1) For each enrolled *relative* of the patient – did they receive a test result (e.g., positive, negative, uncertain) on the Color Quarterly Report (yes/no)? Here we are looking at presence vs. absence of a test result, for each relative. Those with a test result listed will be considered to have completed genetic testing through Color via the GIFT Study.
2) Then, we will sum the number of relatives who completed genetic testing per #1 above for this patient to arrive at the numerator for this outcome – number of enrolled relatives who completed the genetic testing process.

The Family Genetic Testing Fraction is the number of enrolled relatives who obtain a genetic test result (complete the genetic testing process) from Color via the GIFT Study *divided by* the number of relatives reported on the baseline PICS survey of the patient who invited them to the study (see **Appendix B**). The endpoint of interest is the presence (as opposed to the absence) of a test result on the Color Quarterly Report six months after the last relative enrolls in the trial.

We will compare the Family Genetic Testing Fraction for patients by study arm to determine the independent effects of the two virtual platform design features on relatives' receipt of genetic testing. Family Genetic Testing Fraction is being used as the primary study outcome because it is the most inclusive and pragmatic outcome relevant to cascade genetic testing in practice and has high internal validity- that is, it is assessed uniformly across all trial arms.

Secondary Outcomes

Family Invite Fraction: The proportion of each enrolled patient's relatives who are invited to join the study. For each enrolled patient, this will be calculated as the number of invited relatives (assessed via paradata) divided by the number of relatives reported on that patient's baseline PICS survey. We will compare the Family Invite Fraction for patients by study arm to determine the independent effects of the two virtual platform design features on the proportion of relatives invited by

each patient to enroll in the study. This measure will illuminate how the trial features influenced patients' willingness to invite eligible relatives and will be ascertained completely and measured uniformly across all study arms.

Patient assessment of family communication about genetic testing: We will ask patients to assess their capacity, opportunity, and motivation to communicate with family members about their genetic testing results using a 20-item scale we developed for the study. Responses are on a 5-point Likert scale from "not at all true" to "very true." We will assess this measure at two time points: on the baseline PICS survey (see **Appendix B**) and the Patient Six-Month Follow-Up Survey (see **Appendix E**).

The outcome is continuous. We will calculate the mean score at each timepoint and calculate the difference between baseline to follow-up across study arms. The possible scores at each time point range from 1.0 to 5.0. A larger difference between timepoints will indicate greater improvement in the patient's assessment of their communication with relatives.

Relative report of formal genetic risk evaluation**:** We will ask relatives to report on receipt of formal cancer genetic counseling based on a single question: In the past six months, have you had a counseling visit with a genetics expert to discuss cancer risk – that is, an appointment where the whole discussion is about genetic risk for cancer? Relatives will answer on a binary scale (yes/no) and this will be assessed on the Relative Six-Month Follow-up Survey (see **Appendix F**). "Yes" will indicate receipt of formal GRE.

Exploratory Outcomes and Additional Measures

Socioeconomic Status (SES) subgroup analyses: We will explore the above-defined Family Genetic Testing Fraction across SES subgroups using variables derived from SEER clinical/demographic data (e.g., race/ ethnicity, sex, insurance status, and census-level poverty indicator) and the baseline PICS survey (race/ethnicity, income, insurance status, and education) This will be an important assessment of SES gradients in key outcomes across Trial arms to inform whether there were disparities in the impact of the trial among patients and relatives.

Tailoring information: We will collect information needed to tailor or personalize the relative-level intervention, such as whether the relative knew about their inviting patient's cancer history or PV carrier status prior to joining the study; how much they felt they understood the risk of cancer in their family and were interested in testing prior to joining the study; and how interested they are (pre-educational intervention) in getting a genetic testing for cancer risk. This information will be used during the course of the intervention to normalize their current state and encourage them to move through the material and get tested if desired and will be assessed in the Relatives Baseline Survey (see **Appendix D**).

Table 10 summarizes the data sources for each measure described above.

Table 10. Summary of Measures Ordered by Data Source						
	**SEER clinical/ demographic data**	**PICS Survey**	**Platform data (Relatives Baseline Survey/ Paradata)**	**Patient follow-up survey**	**Relative follow-up survey**	**Color Quarterly Report**
**Primary Outcome**						
Family Genetic Testing Fraction		X				X
**Secondary Outcomes**						
Family Invite Fraction		X	X			
Patient assessment of family communication about genetic testing		X		X		
Relative report of formal genetic risk evaluation					X	
**Exploratory Outcomes/Measures**						
SES subgroup analyses	X	X				
Tailoring Information			X			

6 Procedures

6.1 Data Management

<u>SEER Clinical and Demographic Data</u>

Information routinely collected by SEER cancer registries includes demographic and clinical information. To permit SES subgroup analyses, enable the description of participants and non-participants for publications resulting from this population-based study, and comply with clinical trial reporting requirements, the SEER registries will provide Michigan with a de-identified file containing demographic and clinical information along with the Study ID (Access Code); *no patient identifiers (PII) will be included.* This data file will be shared with Michigan via secure file transfer and stored on a secured server (e.g., Dropbox Team Folder). No participant contact is required to obtain this data.

<u>PICS Survey Data</u>
This project begins with a pre-trial paper patient survey designed to assess eligibility for invitation into the trial and collect information needed to assess primary and secondary outcomes (see **Appendix B**). Data will be collected as described in **Section 4.2 Recruitment**. Though follow-up efforts to increase survey response will

cease after the 4-month mark, completed surveys received after this point in time will still be considered for trial eligibility until 4 months after the final batch of surveys is mailed.

As completed paper surveys are received, the SEER field teams at Emory University and USC will perform an initial review of the surveys and remove any identifying information that may have been supplied by the patient (e.g., written into open-ended questions or written in the margins of the survey). This step is taken to protect patient privacy. At this time, the field teams will also review surveys for any missing data and make attempts to call the patients to obtain this information.

SEER teams will ship batches of completed surveys to DataForce, Inc. – the GIFT Study third-party data processing service partner – on a weekly basis. *Note: surveys are de-identified and only a Study ID (Access Code) is used to identify participants.* Prior to shipping, SEER will also scan the surveys to create a PDF copy that will serve as a backup in case a survey shipment is damaged or lost in transit. DataForce will use scanning software to process the surveys and will apply standardized rules agreed upon in advance by the study team re: common data quality issues, such as how to handle multiple marks to single response questions and illegible open-ended fields. All fields that fall out of tolerance (produce errors after the rules are applied) and all open-ended fields will be reviewed by a human operator at DataForce to ensure high-quality data.

DataForce will return the processed survey data back to the UM study team on a routine basis (i.e., weekly or biweekly). Data will be transferred via secure file transfer and stored securely on Michigan Medicine HITS servers (see below for system security details). *Note: surveys remain de-identified and only a Study ID (Access Code) is included in the dataset. UM has no identifiable information about potential trial participants at this stage of the study.* An algorithm will review survey data to determine whether each individual patient is eligible for invitation into the trial. This information will be displayed on the SEER Team administrative dashboard.

Data will remain de-identified until a patient provides informed consent and enrolls into the trial. Upon patient enrollment, selected information from the PICS survey will be used to populate the intervention and tailor (personalize) the content. This avoids the need to ask patients to re-enter information they have already reported to the study via the PICS Survey.

<u>Platform data (Relatives Baseline Survey and Paradata)</u>

Relative baseline survey: Enrolled relatives complete a brief baseline survey immediately upon account creation and before accessing the intervention content (e.g., key facts, etc.) This survey is hosted electronically in UM Qualtrics and seamlessly integrated into the study website experience. It will collect information needed to tailor the relative's intervention website experience (see **Appendix D**).

The GIFT Study website is developed by the University of Michigan Center for Health Communications Research (CHCR). The website applications that CHCR develops are hosted and managed on virtualized servers provided by Michigan Medicine HITS. All servers and the back-end databases (for data storage) are password protected. The server runs the RedHat Linux Enterprise operating system. Security patches and updates are downloaded and installed automatically. Each server is also protected by firewalls to restrict network access to the server. The data collected from participants is stored separately from the application (per Standard Practice Guidelines) on databases also acquired from Michigan Medicine HITS.

Servers and databases used by CHCR includes the safeguards required by HIPAA and may be used to maintain Protected Health Information. The system that CHCR develops goes through an independent security review with Michigan Medicine Information Assurance. This review includes requirements such as: a code review, vulnerability scans, and penetration testing (to determine if there are any hackable risks).

When a participant accesses the GIFT study website, content will be transmitted securely using the Transport Layer Security (TLS) protocol, the same protocol used to protect financial and other personal information when transmitted from a web site to a user's browser. This prevents anyone else on the network from intercepting and viewing the content that is being provided by or to the participant.

The intervention platform will collect survey data from patient and relative participants throughout their use of the website. This will include:

- Patient contact information, entered during account creation
- Relative contact information, entered by patients during the invitation process
- Relative eligibility survey data
- Relative contact information, entered during account creation
- Relative baseline survey data

Paradata: The website will also track and record paradata such as user actions (clicks) and time spent on each webpage. In addition to this traditional paradata, the website will also record:

- Number of patients enrolled
- Number of relatives invited by patients (needed for Secondary Outcome: Family Invite Fraction)
- Number of invited relatives who are eligible for the GIFT study after screening
- Number of relatives enrolled
- Number of relatives who initiate genetic testing (click the button to go to Color Health's website)

Data Access and Transfer. The GIFT study team will require access to the participant data collected by the platform. All data will be available via a password-protected project dashboard built and administered by CHCR team members. Different access roles will be defined and study team access will be provided on a "need to know" basis. Michigan study staff will have full access; Stanford study staff will have access to enrolled patients and relatives in Study Arms 3&4; Emory and USC SEER teams will only be able to view their site-specific patient eligibility and enrollment status (in order to facilitate follow-up efforts to encourage trial enrollment). Eligibility, enrollment, and study progress can be viewed directly on the dashboard, and more detailed data files will be available for download by study team members from the site as .csv files.

<u>Patient and Relative Six-Month Follow-Up Surveys</u>

Follow-Up Surveys will be hosted via UM Qualtrics and patients and relatives will be asked to complete the applicable survey six months after their enrollment in the GIFT Study RCT. Participants will be invited via email to complete the electronic survey in their preferred language (English or Spanish). The GIFT study platform will send participants an email written at the 7th-8th grade reading level that consists of 1) a link to complete the electronic survey; 2) a telephone number to call with any questions or concerns; 3) a promise of a $20 electronic gift card upon survey

completion. We will employ a modified version of the Dillman method to enhance response rates and will follow up with both automatic and manual email reminders, telephone calls, and/or text messages to answer questions and provide assistance as needed to ensure optimal response rates on the following schedule:

- Days 3, 7, 14, 21, 30, and every 2 weeks thereafter until 7-month mark

Follow-up efforts for encouraging survey completion will continue for 7 months, because in our team's prior experience it can often take this amount of time to exhaust opportunities for encouraging response. Follow-up efforts will cease if a direct refusal or completed survey are obtained.

Data collection of the follow-up surveys will be completed via UM Qualtrics. Data will then be securely stored on Michigan Medicine HITS databases with the rest of the GIFT study data where it can be accessed by the study team through their staff dashboard post-authentication. The Biostatistican(s) will conduct data cleaning prior to analysis.

Color Quarterly Report

Each quarter, Color will provide the Michigan study team with a de-identified quarterly report in excel (.xlsx) via secure file transfer. The data will contain no PII and will be stored on a secure, access-limited UM Dropbox team folder. The report will contain, by Color token ID (unique identifier; each enrolled relative has one), information regarding the last reported: testing status, test result (and if positive, affected gene), and other key information to help the team understand the primary outcome – whether or not each enrolled relative completed genetic testing via Color through the GIFT Study.

No participant contact is required to obtain this data. Reports will be snapshots in time and will allow the study team to monitor uptake of the intervention and downstream intervention effects (testing). The final quarterly report received six months after the final relative enrolls in the study will be merged with the other analytic datasets for analysis.

Analytic Dataset Creation

All data collected during the study as described above will be merged using the unique Study IDs (Access Codes) for patients and relatives to create the analytic datasets. These datasets will be stored and maintained by the biostatistician team in a U-M Dropbox Team Folder approved for storage of data containing PHI.

Data Quality Monitoring

The Biostatistician will assess the PICS survey data (the only survey that will be made available in paper format) for the degree of missing values on an ongoing basis during the initial patient survey fieldwork. Otherwise, we anticipate minimal missing data from that collected online, based on the prior expertise of the study team in conducting and collecting online data.

PICS Surveys are handled via DataForce, a third-party data processing service. DataForce will be required to set ranges on write-in survey questions, where applicable, in order to capture erroneous data before it is written to the data file. If values fall outside of expected ranges, a human operator will review the field to

check for substitution errors. All multiple marks must be reviewed by a human operator to see if respondent intended one response over the other. The study team will provide DataForce with rules for how to handle common data errors such as multiple marks, illegible text, etc. DataForce implements several quality checks throughout their process that include reviews from supervisors, the technology team, and the project management team. This results in at least 5% of the forms being thoroughly checked for quality. If inconsistencies are found between operators, all forms processed by the operator with errors will be reprocessed. Data scanning accuracy is close to 99.9% and manual key entry (of open-ended fields) averages 96-98% with single entry and 99.9% with double key entry.

7 Data Analysis

7.1 Analysis Plan

We will first examine descriptive statistics for all variables of interest to assess data quality and look for potential outliers. As described above, we are testing the main effects of two interventions, represented by the separate platform design features of reduced vs free test cost and web-based vs human navigator communication and decision support. The 2x2 factorial design with block randomization at the level of the index patient (j) will result in four groups of index patients of equal size. The relatives (i) will be offered the same intervention as the index patient who invited them and the design is considered cluster randomized for analyses that include relatives. The aims vary in terms of the target of the intervention and the outcomes measured.

There are three general issues relevant to these analyses. First, we do not hypothesize that there will be any synergistic effect of the two treatments on the log odds scale and thus assume that the effects will be additive. Conceptually we cannot think of any reason that the effect of the cost of the testing and any help provided by a navigator over the website have an obvious reason to be dependent on the level of the other. Thus, our primary outcome is the treatment effect of each intervention in a regression model without an interaction term.

The second analytic issue is that of informative cluster size. An assumption of the generalized linear multilevel models used in our analysis is that cluster size is not informative. The literature on family communication does not generally talk about family size as one of the factors that predicts health communication but there is little data one way or the other looking specifically at genetic testing. Thus, there is little to guide us on whether larger families are more or less likely to communicate well, or be likely to invite relatives, or whether relatives in larger families are more or less likely to get genetic testing when there is a potential health benefit. Thus, given the conditional independence assumption we will condition the models on cluster size with a threshold of including this covariate as a p value of 0.10 or less.

Finally, some of our analytic models will have other covariates including cluster size noted above and in some cases baseline levels of the outcome variable for models estimating change from a baseline variable. All of our models will include the stratification design variable for site (CA vs GA). In all models that include covariates we will estimate the marginal main effect of each treatment by averaging over the distribution of the covariates included in each model (such as baseline levels of outcome variables, state or cluster size). This in effect standardizes the treatment effect to a population with the distribution of those covariates observed in our study sample. For generalization purposes we have a fully population-based sample drawn from SEER registries. Thus, the baseline distribution of non-treatment covariates in our study sample is directly generalizable to the index patient population of the two large states from which the sample is drawn who are willing to enroll in a program to increase informed family participation in genetic testing. This is the target population of interest for any broader program that could be developed if the trial is successful

For the **Primary Aim (Family Genetic Testing Fraction),** the dependent variable is the number of relatives who complete genetic testing from Color using a study-provided token. **This outcome is the pre-specified primary outcome of the trial.** We will describe a "*Family Genetic Testing Fraction*" as the proportion of eligible relatives of a patient who complete genetic testing. We anticipate that zero cost of the test and increased navigation support will be associated with a greater proportion of relatives completing testing, as quantified by the coefficients and standard errors of these covariates. For the Primary Aim, the target population is all eligible relatives who are identified by the index subject. The intervention is randomized by index patient. We approach relatives only through the index patient, who will have an influence on the relatives' responses based at a minimum on their prior relationship and most likely with direct communication about the study. Thus, the analysis for this Primary Aim will account for the cluster level randomization of the intervention and nesting of relatives within each index patient using multilevel generalized linear models. We will use a multilevel binomial regression model with a random intercept estimated for each index patient, the number of successes (relatives who are tested) as the outcome and the number of trials set as the number of eligible relatives. We will include the number of eligible relatives as a covariate in the model if the coefficient is significant (at the p=0.10 level). We will also include relative sex and the patient-reported assessment of communication with family scale score to improve power as these baseline variables are likely to predict the outcome. No other variables will be included. The main effects of each intervention will be quantified by the marginal effect of the respective treatment variable averaging across the other treatment variable, covariates and cluster (eligible family) size.

For **Secondary Aim 1 (Family Invite Fraction),** the dependent variable is the number of a patient's total eligible relatives for whom the patient is willing to provide contact information given the total number of eligible relatives ("*Family Invite Fraction*"). We expect that zero cost and increased navigation help will be associated with an increase in the proportion of relatives for whom a patient is willing to provide contact information, quantified by the coefficients and standard errors of these variables. We will use a multilevel binomial regression model with a random intercept estimated for each index patient, the number of successes (family members for whom an address is provided) as the outcome and the number of trials set as the number of eligible relatives. We will include the number of eligible relatives as a covariate in the model if the coefficient is significant (at the p=0.10 level). We will also consider including the scale score of patient report of assessment of communication with family collected on the baseline PICS survey to improve power. No other variables will be included. The main effects of each intervention will be quantified by the marginal effect of each treatment variable averaging across the included covariates.

For **Secondary Aim 2 (Assessment of Family Communication Scale),** the target population of the intervention is the index cancer patient. The analytic sample includes all patients who consent and are randomized (expected n = 880). The dependent variable is the patient's change from baseline in their assessment of their communication with relatives ("*Patient assessment of family communication about genetic testing")*, which will be measured as a continuous outcome using a multi-item scale. We anticipate that increases in mean assessment score will be associated with zero cost and genetic risk navigation help. We will use linear regression to estimate the main effects of cost reduction and navigator help included as two treatment (1/0) variables. Each index patient's baseline assessment score will be included as a covariate. We will include cluster size as a covariate if the coefficient is significant at the 0.10 level. The main effects of each intervention will be quantified by the marginal effect of each treatment variable averaging across the included covariates. The standard error of the main effect will define the confidence interval for the intervention effect.

Secondary Aim 3 (Relative Receipt of Formal Genetic Risk Evaluation) involves analyses of enrolled relatives' report of formal genetic risk evaluation within the past six months. While index patients and their 1st and 2nd degree relatives are randomized, the enrolled relatives result from some unrandomized factors, specifically the differential effect of the intervention across randomization groups on invitation and enrollment. The larger the effect of the primary

intervention, the less comparable the groups of enrolled relatives will be based on the original cluster randomization. Thus, the analyses for this secondary aim 3 will be labeled as observational and we will attempt to control for factors that predict ultimate enrollment in the trial. Outcomes will still be analyzed using a multilevel linear regression model with a random intercept estimated for each index patient to account for clustering by index patient. For Secondary Aim 3 the analytic sample includes all relatives who are invited by eligible patients and consent (anticipated N = 1,584). The intervention is randomized by index patient and we approach relatives only through the index patient, who will have an influence on the relatives' responses based at a minimum on their prior relationship and most likely with direct communication about the study. The analyses for these aims thus will account for the cluster level randomization of the intervention and nesting of relatives within each index patient using multilevel generalized linear models.

As an **exploratory analysis**, we will examine the effect of the two virtual platform design features on the Primary Outcome (Family Genetic Testing Fraction) and Secondary Outcome 1 (Family Invite Fraction) across patient socioeconomic (SES) characteristics. These will be measured with variable for patient race/ethnicity, insurance status, and census tract level poverty rate. Starting with the multilevel binomial regression models used for the primary and secondary aim 1 analyses, we will include the SES variables as covariates in each model. We will examine the marginal effects of the treatment variables in each model, stratified by each of the SES variables. We will further explore the extent to which SES modifies the main effects by testing for interactions between the SES and main effects in each model. For all models we will examine residual plots from the models to assess model assumptions. We will also carry out sensitivity analyses to assess for an interaction between the two trial features and any effects of time period (by 6-month periods).

7.2 **Power Considerations**

The primary outcome of the study is the proportion of relatives who complete genetic testing as described above and our sample size calculations focus on that analysis.
Assumptions: Effect sizes for the intervention are proposed to be the minimum clinically significant and achievable effect for assumed testing rates varying by trial features as shown in **Table 11**.

Table 11: Minimally detectable absolute difference between study arms

	No Navigator	Navigator
$50	10%	15%
Free	13%	18%

These probabilities reflect odds ratios of 1.4-1.5 which were used as the middle of the range modeled in the sample size calculations. To account for clustering in the relatives' outcomes at the patient level, we assume an estimate of 0.15 for the residual intra-class correlation (after accounting for the intervention effects). We anticipate enrolling 880 index cancer patients with a median of 8 eligible relatives. With these assumptions, a simulation based on our planned analytic approach confirms a power of at least 0.80 for the effect of zero cost of the genetic test and navigation support for any effect including or above these specified effect sizes.

The estimates will be more precise, and the power will be higher for secondary aim 1 (family invite fraction) as the proportion of relatives invited will be higher than those who get tested with the same denominator, as well as for the continuous outcome of secondary aim 2 (patient assessment of family communication) than for either of the binary outcomes.

8 Protection of Human Subjects

This research study has an excellent risk/benefit ratio. While risks to participants are minimal, the benefits to the participating patients and relatives are great. There is also potential benefit to future families via knowledge gained and potential improvements to care quality.

Potential Benefits

Participation in this study provides direct benefit for enrolled patients and relatives. Patients benefit by receiving help talking with their family members about hereditary cancer risk and genetic risk evaluation – a topic that is often emotionally challenging. Relatives benefit by receiving personalized education regarding their familial hereditary cancer risk and the benefits of genetic risk evaluation and testing for their future health. Relatives also benefit via their ability to obtain medical genetic testing for cancer risk for free or minimal cost ($50), depending on their study arm. Such testing normally costs at least $250 and often much more, particularly for second-degree relatives or for those seeking testing months or years out from the initial variant being found to run in the family. Finally, both patients and relatives in Arms 3 & 4 (the Family Health Navigator arms) may benefit from an additional human source of support throughout the family communication and genetic testing experience.

More broadly, this study has the potential to benefit future patients and families by driving effective cancer prevention and informing cascade genetic testing delivery among cancer patients and their relatives. This work has tremendous potential to reduce the burden of cancer at the population level.

Compensation:

- Patients receive an up-front $20 cash or gift card incentive with their PICS Survey, which they may keep regardless of whether or not they return a completed survey. Patients also receive a $20 electronic gift card upon completion of their six-month follow-up survey. Total compensation for enrolled patients in the study is $40.
- Relatives receive a $20 electronic gift card upon completion of their six-month follow-up survey.
- Note: as this is designed to be a pragmatic trial, RCT enrollment for patients and relatives is not incentivized or compensated in any way.

Potential Risks

We have crafted a study strategy to minimize risk to patients and their relatives. Our study team uses established human subjects protection protocols that address the limited use of PHI and strict protection of these data, transfer of de-identified datasets within and between institutions, storage of the information from various data sources, and surveillance and response to adverse events. A key component of our strategy is bi-weekly staff meetings and between-institution investigators and staff conference calls to review, update, and monitor all study activities. Study participants and potential participants are provided with phone numbers and email addresses to contact the study team with any questions or concerns about the study at every step in the study protocol.

The primary potential risk to both patients and relatives is breach of confidentiality of personally identifiable information (PII) and/or protected health information (PHI). This risk is low, given the strategies the research team will

implement to minimize risk of breach of confidentiality to patients and relatives. To protect participants from the risk of breach of confidentiality, the study team will take the following steps: 1) Study team members will maintain protection of human subjects training and the staff involved will be restricted to the minimum number necessary to conduct the work. 2) IRB approvals will be secured and data use agreements will be put in place where required before any data sharing occurs and shared data will be kept to the minimum required to accomplish the work. 3) All data will be transmitted using encryption techniques and stored securely on password-protected servers. 4) Data will be de-identified before being shared between study team members whenever feasible. 5) All PII collected from patient and relatives trial participants that is needed to deliver the services offered in this study will be destroyed at the conclusion of the study and only anonymous identifiers will be retained for analysis. 6) The UM data analytic team will receive only de-identified datasets as defined in HIPAA privacy regulations. 7) The study will utilize a third-party honest broker (IMS) to sample the inception patient cohort and construct the final patient-level analytic dataset (please see Section 4 Methods and Section 6 Procedures for details).

A second potential risk is negative patient or relative reactions to study procedures, including 1) survey questions about cancer, health care experiences, and experiences with GRE; 2) the offer presented to patients to provide contact information and invite their relatives to participate in the trial; and 3) the ability for enrolled relatives to order genetic testing. We will address these risk as follows:

1) Our study team has had a very strong track record of positive engagement with patients and families for many years with very high survey response rates and minimal negative reaction. This is due to the support patients and families have for the mission of our science to improve health outcomes, the quality of the research team, the quality of the content of our materials, and the thoughtful procedures we deploy to engage potential participants in a highly respectful and confidential manner. We are particularly proud of our track record of strong support from vulnerable populations including racial and ethnic minorities, non-native English speakers, and sicker patients and expect the risk of negative patient and relative reactions to survey materials to be low. As in our prior SEER-registry based survey research, all surveys will be kept as brief as possible to minimize inconvenience. Survey participation (and study participation in general) is voluntary and participants may choose to skip questions or stop participation at any time or decline participation altogether. This will be explained up front to all participants in the informed consent documentation and the study team will follow up with any participants who contact the study team with concerns or negative reactions to provide support and resources.

2) All tasks or actions available to participants in the intervention are optional. We recognize that some patients may not want to invite their relatives to participate and thus the study consent and platform will explain that this step, like every other available task, is voluntary. We will offer the trial to relatives only through patient voluntary release of relative contact information, consistent with the standard of US clinical practice for cascade GRE. This will be explained up front to the relative in the informed consent document and the study team will follow up with any participants who contact the study team with concerns or negative reactions to provide support and resources.

3) Finally, relatives may experience anxiety before or after their use of the optional genetic testing service provided for the study by Color Health. *Pre-test* anxiety is not expected to be any different from that experienced by the

population outside of the research context. Relatives who opt to receive genetic testing through the study will undergo Color's standard protocol for pre-test informed consent and Color provides a customer support telephone line for questions and concerns. The intervention normalizes the experience of anxiety as natural and provides reassurances that the information they will gain from testing will be helpful for cancer screening and risk reduction decision-making. Relatives in Arms 3 & 4 will also have access to the Family Health Navigator for additional support.

The Michigan and Stanford staff teams (Family Health Navigator) will have real-time access to the Color Health Provider Portal (CPP), which will allow the study team to provide participant support during the test ordering process for relatives. The CPP displays individual level information for relatives who order genetic testing through the GIFT Study, including PII, testing status, and test results (when available). *The study team will access the CPP only upon receipt of a participant question or request for support*; routine monitoring will not occur and data exports are not available. Access to the CPP will be limited to only those staff with a study-related need to know. The study team will not provide post-test counseling and will direct participants who reach out with such needs to contact the Color Health genetic counseling team and/or their personal physician. The CPP is available to Michigan and Stanford staff for purposes of providing participant help and support during the test ordering process only.

Post-test difficulties in dealing with genetic test results may be experienced by some enrolled relatives, particularly if their test result is positive for a PV that increases cancer risk. Again, these negative or anxious reactions are not expected to be any different from those experienced by the population outside of the research context. Relatives who receive genetic testing from Color will undergo Color's standard protocol for results reporting through the company, which includes required genetic counseling for those with identified PVs (with optional genetic counseling available for everyone else as well). As indicated above, the GIFT Study does not provide post-test counseling for enrolled relatives and we will direct relatives who request this to Color or to their regular treating physician(s).

To further manage the risk of negative emotional reactions to testing, we will query relative participants in the relative participant follow-up survey six months after trial enrollment and the Stanford Clinical Genetics Center faculty and staff and/or the Family Health Navigator will respond to participants who report strong negative reactions to test results, regardless of study arm. Based on our prior research[4] we expect this risk to be low, with fewer than 10% of relatives reporting significant difficulties coping with genetic testing results.

9 Data and Safety Monitoring

This study will be monitored in accordance with the NCI approved University of Michigan Rogel Cancer Center Data and Safety Monitoring Plan.

The study team will meet every six months or more frequently depending on the activity of the protocol. The discussion will include matters related to the safety of study participants (SAE/UaP reporting), validity and integrity of the data, enrollment rate relative to expectations, characteristics of participants, retention of participants, adherence to the protocol (potential or real protocol deviations) and data completeness. At these regular meetings, the protocol specific Data and Safety Monitoring Report form will be completed and signed by the Principal

Investigator or by one of the co-investigators.

Data and Safety Monitoring Reports will be submitted to the University of Michigan Rogel Cancer Center Data and Safety Monitoring Committee (DSMC) every six months for independent review.

10 Adverse Event Reporting

This study is designed to minimize risk to patients and their relatives. Study participants (or potential participants) who need support or want further clarification of any issue will be contacted by the appropriate investigator (SEER, Stanford, or Michigan depending on the issue) in a sensitive and professional manner. Participants who reach out for further information or have concerns about any aspects of their cancer care will be advised to speak to their treating physicians. We will also make available regional and national resources for information about GRE and management such as *The National Society of Genetic Counselors, the Facing Our Risk of Cancer Empowered (FORCE) group, and the Georgia Center for Oncology Research and Education (CORE)* websites.

Adverse events related to this study are not expected. However, any adverse events resulting from research procedures will be reported to IRBMED per institutional guidelines. Should any adverse events appear to be occurring frequently, we will amend the protocol to address these concerns. We do not anticipate having to stop the trial for these types of adverse events should they occur.

11 References

1. Offit K, Tkachuk KA, Stadler ZK, et al. Cascading After Peridiagnostic Cancer Genetic Testing: An Alternative to Population-Based Screening. *Journal of clinical oncology : official journal of the American Society of Clinical Oncology.* 2020;0(0):JCO.19.02010.
2. Ladabaum U, Wang G, Terdiman J, et al. Strategies to identify the Lynch syndrome among patients with colorectal cancer: a cost-effectiveness analysis. *Ann Intern Med.* 2011;155(2):69-79.
3. Owens DK, Davidson KW, Krist AH, et al. Risk Assessment, Genetic Counseling, and Genetic Testing for BRCA-Related Cancer: US Preventive Services Task Force Recommendation Statement. *Jama.* 2019;322(7):652-665.
4. Kurian AW, Ward KC, Hamilton AS, et al. Uptake, results, and outcomes of germline multiple-gene sequencing after diagnosis of breast cancer. *JAMA oncology.* 2018;4(8):1066-1072.
5. Kurian AW, Katz SJ. The Emerging Opportunity of Cascade Genetic Testing for Population-Wide Cancer Prevention and Control. *Journal of clinical oncology : official journal of the American Society of Clinical Oncology.* 2020;In Press.
6. Giri VN, Hegarty SE, Hyatt C, et al. Germline genetic testing for inherited prostate cancer in practice: Implications for genetic testing, precision therapy, and cascade testing. *Prostate.* 2019;79(4):333-339.
7. Hattinger CM, Patrizio MP, Tavanti E, et al. Genetic testing for high-grade osteosarcoma: a guide for future tailored treatments? *Expert Rev Mol Diagn.* 2018;18(11):947-961.
8. Chung DC. Genetic Testing and Early Onset Colon Cancer. *Gastroenterology.* 2018;154(4):788-789.
9. Lorans M, Dow E, Macrae FA, Winship IM, Buchanan DD. Update on Hereditary Colorectal Cancer: Improving the Clinical Utility of Multigene Panel Testing. *Clin Colorectal Cancer.* 2018;17(2):e293-e305.

10. Stoffel EM, McKernin SE, Brand R, et al. Evaluating Susceptibility to Pancreatic Cancer: ASCO Provisional Clinical Opinion. *Journal of clinical oncology : official journal of the American Society of Clinical Oncology.* 2019;37(2):153-164.
11. Wood LD, Yurgelun MB, Goggins MG. Genetics of Familial and Sporadic Pancreatic Cancer. *Gastroenterology.* 2019;156(7):2041-2055.
12. Daly MB, Pilarski R, Berry M, et al. NCCN Guidelines Insights: Genetic/Familial High-Risk Assessment: Breast and Ovarian, Version 2.2017. *J Natl Compr Canc Netw.* 2017;15(1):9-20.
13. Gupta S, Provenzale D, Regenbogen SE, et al. NCCN Guidelines Insights: Genetic/Familial High-Risk Assessment: Colorectal, Version 3.2017. *J Natl Compr Canc Netw.* 2017;15(12):1465-1475.
14. Richards S, Aziz N, Bale S, et al. Standards and guidelines for the interpretation of sequence variants: a joint consensus recommendation of the American College of Medical Genetics and Genomics and the Association for Molecular Pathology. *Genetics in medicine : official journal of the American College of Medical Genetics.* 2015;17(5):405-424.
15. Caswell-Jin JL, Zimmer AD, Stedden W, Kingham KE, Zhou AY, Kurian AW. Cascade Genetic Testing of Relatives for Hereditary Cancer Risk: Results of an Online Initiative. *J Natl Cancer Inst.* 2019;111(1):95-98.
16. Idos GE, Kurian AW, Ricker C, et al. Multicenter Prospective Cohort Study of the Diagnostic Yield and Patient Experience of Multiplex Gene Panel Testing For Hereditary Cancer Risk. *JCO Precision Oncology.* 2019(3):1-12.
17. Ricker CN, Koff RB, Qu C, et al. Patient communication of cancer genetic test results in a diverse population. *Transl Behav Med.* 2018;8(1):85-94.
18. Sanghera C, Sanghera R. Immunotherapy - Strategies for Expanding Its Role in the Treatment of All Major Tumor Sites. *Cureus.* 2019;11(10):e5938.
19. National Cancer Institute. *Precision Prevention and Early Detection Working Group Recommendation: Cancer Prevention and Early Detection in Individuals at High Risk for Cancer.* 2019.
20. Kurian AW, Ward KC, Abrahamse P, et al. Association of Germline Genetic Testing Results With Locoregional and Systemic Therapy in Patients With Breast Cancer. *JAMA oncology.* 2020.
21. Katz SJ, Ward KC, Hamilton AS, Abrahamse P, Hawley ST, Kurian AW. Association of Germline Genetic Test Type and Results With Patient Cancer Worry After Diagnosis of Breast Cancer. *JCO Precision Oncology.* 2018(2):1-8.
22. Katz SJ, Ward KC, Hamilton AS, et al. Gaps in Receipt of Clinically Indicated Genetic Counseling After Diagnosis of Breast Cancer. *Journal of clinical oncology : official journal of the American Society of Clinical Oncology.* 2018:JCO.2017.2076.2369.
23. Kurian AW, Ward KC, Howlader N, et al. Genetic Testing and Results in a Population-Based Cohort of Breast Cancer Patients and Ovarian Cancer Patients. *Journal of clinical oncology : official journal of the American Society of Clinical Oncology.* 2019;37(15):1305-1315.

12 Appendices

Appendix A: Abbreviations

ARR	At Risk Relatives
CORE	Georgia Center for Oncology Research and Education
CPP	Color Health Provider Portal
DSMB	Data and Safety Monitoring Board
FORCE	Facing Our Risk of Cancer Empowered
GIFT	Genetic Information and Family Testing
GRE	Genetic Risk Evaluation
HCS	Hereditary Cancer Susceptibility
IMS	Information Management Services Inc.
MGP	Multigene Panel
NAACCR	North American Association of Central Cancer Registries
NCI	National Cancer Institute
NHIA	NAACCR Hispanic Identification Algorithm
PHI	Protected Health Information
PICS	Patient Inception Cohort Survey
PV	Pathogenic Variant
RCT	Randomized Controlled Trial
SEER	Surveillance, Epidemiology, and End Results Program
SES	Socioeconomic Status
UM	University of Michigan

Appendix B: PICS Survey

Note: Headings and citation information for protocol purposes only and will not be presented to participants during the survey.

English Version

Trial Eligibility Information

Have you ever had a blood or saliva genetic test for cancer risk that was ordered by a doctor or genetics expert?

○ **Yes** The results were **positive** I have a gene mutation that increases the risk of cancer	○ **Yes** The results were **uncertain** A variant of uncertain significance (VUS) was found	○ **Yes** The results were **negative** I do not have any gene mutations	○ **No** I have never had a cancer genetic test

Must answer "yes – the results were positive" in order to be considered eligible for the trial.

Katz SJ, Ward KC, Hamilton AS, Mcleod MC, Wallner LP, Morrow M, Jagsi R, Hawley ST, Kurian AW. Gaps in Receipt of Clinically Indicated Genetic Counseling After Diagnosis of Breast Cancer. J Clin Oncol. 2018 Apr 20;36(12):1218-1224. doi: 10.1200/JCO.2017.76.2369. Epub 2018 Mar 12. PMID: 29528794; PMCID: PMC5908222.

Family Members
Items created de novo for this study

Your biological mother
Is your **biological mother** alive today and living in the USA, Canada, or Mexico?

○ Yes
○ No ⟶ Skip to Your Maternal Aunts – Mother's Sisters

Your Maternal Aunts (Mother's Sisters)
Next, tell us about your **biological aunts** ages 18 and up and living at the time of this survey in either the USA, Canada, or Mexico.

> **A maternal aunt is any biological sister of your biological mother**. This does not include an aunt who married into your family.

How many do you have? 0 1 2 3 4 5 6 7 8 9 10 If more than 10, please write in the number here: _____

Your Maternal Uncles (Mother's Brothers)
Next, tell us about your **biological uncles** ages 18 and up and living at the time of this survey in either the USA, Canada, or Mexico.

A maternal uncle is any biological brother of your biological mother. This does not include an uncle who married into your family.

How many do you have? 0 1 2 3 4 5 6 7 8 9 10 If more than 10, please write in the number here: _____

Your biological father
Is your **biological father** alive today and living in the USA, Canada, or Mexico?
- ○ Yes
- ○ No ⟶ Skip to Your Paternal Aunts – Father's Sisters

Your Paternal Aunts (Father's Sisters)
Next, tell us about your **biological aunts** ages 18 and up and living at the time of this survey in either the USA, Canada, or Mexico.

A paternal aunt is any biological sister of your biological father. This does not include an aunt who married into your family.

How many do you have? 0 1 2 3 4 5 6 7 8 9 10 If more than 10, please write in the number here: _____

Your Paternal Uncles (Father's Brothers)
Next, tell us about your **biological uncles** ages 18 and up and living at the time of this survey in either the USA, Canada, or Mexico.

A paternal uncle is any biological brother of your biological father. This does not include an uncle who married into your family.

How many do you have? 0 1 2 3 4 5 6 7 8 9 10 If more than 10, please write in the number here: _____

Your sisters
Next, tell us about your **biological sisters** ages 18 and up and living at the time of this survey in either the USA, Canada, or Mexico.

A biological sister is a sister with the same biological mother and/or father as you. This includes half-sisters with whom you share only one parent. Please do not include stepsisters, adopted sisters, or sisters-in-law.

How many do you have? 0 1 2 3 4 5 6 7 8 9 10 If more than 10, please write in the number here: _____

Your brothers
Next, tell us about your **biological brothers** ages 18 and up and living at the time of this survey in either the USA, Canada, or Mexico.

A biological brother is a brother with the same biological mother and/or father as you. This includes half-brothers with whom you share only one parent. Please do not include stepbrothers, adopted brothers, or brothers-in-law.

How many do you have? 0 1 2 3 4 5 6 7 8 9 10 If more than 10, please write in the number here: _____

Your Daughters
Please tell us about your **biological daughters** ages 18 and up and living at the time of this survey in either the USA, Canada, or Mexico.

A biological daughter is a daughter who was conceived by and born to you. Please do not include stepdaughters, adopted daughters, or daughters-in-law.

How many do you have? 0 1 2 3 4 5 6 7 8 9 10 If more than 10, please write in the number here: _____

Your Sons
Please tell us about your **biological sons** ages 18 and up and living at the time of this survey in either the USA, Canada, or Mexico.

A biological son is a son who was conceived by and born to you. Please do not include stepsons, adopted sons, or sons-in-law.

How many do you have? 0 1 2 3 4 5 6 7 8 9 10 If more than 10, please write in the number here: _____

Your Nieces (the daughters of your sisters and brothers)
Please tell us about your **biological nieces** ages 18 and up and living at the time of this survey in either the USA, Canada, or Mexico.

A biological niece is the biological daughter of your biological brother or sister. Please do not include nieces not conceived by and born to your brothers or sisters, such as their adopted children or stepchildren by marriage.

How many do you have? 0 1 2 3 4 5 6 7 8 9 10 If more than 10, please write in the number here: _____

Your nephews (the sons of your sisters and brothers)
Please tell us about your **biological nephews** ages 18 and up and living at the time of this survey in either the USA, Canada, or Mexico.

A biological nephew is the biological son of your biological brother or sister. Please do not include nephews not conceived by and born to your brothers or sisters, such as their adopted children or stepchildren by marriage.

How many do you have? 0 1 2 3 4 5 6 7 8 9 10 If more than 10, please write in the number here: _____

SES Measures

Which of the following best describes your **current** employment status? **Please mark ALL that apply.**

- ○ Working for pay full-time (40+ hours/week)
- ○ Working for pay part-time (less than 40 hours/week)
- ○ Unemployed and looking for work
- ○ Temporarily laid off or on sick or other leave
- ○ Disabled
- ○ Retired
- ○ Student
- ○ Homemaker

What type of medical insurance do you currently have? **Please mark ALL that apply.**

- ○ None
- ○ Insurance provided through my current or former employer or union (including HMO)
- ○ Insurance provided to another family member (e.g., spouse) through their current or former employer or union (including HMO)
- ○ Insurance purchased directly from an insurance company (by you or another family member)
- ○ Insurance purchased from an exchange ("Obamacare" or the Affordable Care Act)
- ○ Medicaid or other state provided insurance (such as Medi-Cal)
- ○ Medicare/government insurance
- ○ Indian Health Service
- ○ TRICARE or other military health care
- ○ Veterans Affairs (VA, including those who have ever used or enrolled for VA health care)
- ○ Other (please explain): _

Currently, what is the total yearly income of your entire household, before taxes, from all sources – including child support, alimony, disability, social security, and unemployment?

- ○ Less than $5,000
- ○ $5,000-$9,999
- ○ $10,000-$19,999
- ○ $20,000-$29,999
- ○ $30,000-$39,999
- ○ $40,000-$59,999
- ○ $60,000-$89,999
- ○ $90,000-$119,999
- ○ $120,000 or more
- ○ Don't know

Currently, how many people are supported by the total income for your household, including yourself?

○ 1 (just you) ○ 2 people ○ 3 people ○ 4 or more people

What is the highest level of education you have completed?

- ○ No high school
- ○ Some high school
- ○ High school graduate or G.E.D.
- ○ Some college or technical school
- ○ College graduate (Bachelor's degree)
- ○ Graduate degree or higher

What sex were you assigned at birth, meaning on your original birth certificate?

- ○ Female
- ○ Male
- ○ Intersex

Which of the following best describes your race? **Please mark ALL that apply**.

- ○ White
- ○ Black or African-American
- ○ American Indian or Alaska Native
- ○ Native Hawaiian or other Pacific Islander
- ○ Asian Indian
- ○ Chinese
- ○ Filipino
- ○ Japanese
- ○ Korean
- ○ Vietnamese
- ○ Other Asian (please explain): ____________________
- ○ Other Race (please explain): ____________________

Are you of Hispanic, Latino, or Spanish origin?

- ○ Yes, Mexican, Mexican-American, or Chicano
- ○ Yes, Puerto Rican
- ○ Yes, Cuban
- ○ Yes, another Hispanic, Latino, or Spanish origin (please explain): ____________________
- ○ No

Items selected from prior work by our team:

Jagsi R, Abrahamse PH, Lee KL, Wallner LP, Janz NK, Hamilton AS, Ward KC, Morrow M, Kurian AW, Friese CR, Hawley ST, Katz SJ. Treatment decisions and employment of breast cancer patients: Results of a population-based survey. Cancer. 2017 Dec 15;123(24):4791-4799. doi: 10.1002/cncr.30959. Epub 2017 Oct 9. PMID: 28990155; PMCID: PMC5716845.

Hawley ST, Griffith KA, Hamilton AS, Ward KC, Morrow M, Janz NK, Katz SJ, Jagsi R. The association between patient attitudes and values and the strength of consideration for contralateral prophylactic mastectomy in a population-based sample of breast cancer patients. Cancer. 2017 Dec 1;123(23):4547-4555. doi: 10.1002/cncr.30924. Epub 2017 Aug 15. PMID: 28810062; PMCID: PMC5907487.

Assessment of Family Communication Scale

Scale created de novo for this study

I2. Thinking about talking with your biological family about genetic testing for cancer risk, how true are each of the statements below?

	Not at all true	A little true	Somewhat true	Quite true	Very true
a. I understand my genetic test results well enough to talk about them with my family members	○	○	○	○	○
b. I am comfortable talking to my family members about my genetic test results	○	○	○	○	○
c. I am confident that I can talk with my family members about genetic testing	○	○	○	○	○

d. I have enough time to reach out to my family members and talk with them about genetic testing	○	○	○	○	○
e. My family is comfortable talking about genetic testing for cancer risk	○	○	○	○	○
f. I can handle the emotions of my family members when talking about genetic testing	○	○	○	○	○
g. I can handle my own emotions when talking with my family members about genetic testing	○	○	○	○	○
h. I don't know which family members I should talk with about genetic testing for cancer risk	○	○	○	○	○
i. I feel as though there's nothing more I can do to encourage my family members to get genetic testing	○	○	○	○	○
j. My family members have asked me about my genetic test results	○	○	○	○	○
k. I believe that genetic testing can help prevent cancer in my family members	○	○	○	○	○
l. I want my family members to get genetic testing	○	○	○	○	○
m. A health care provider or genetic counselor encouraged me to share my genetic test results with my family members	○	○	○	○	○
n. I believe that my genetic test results are useful to my family members	○	○	○	○	○
o. I feel that it is my responsibility to share my genetic test results with my family members	○	○	○	○	○
p. I believe that sharing my genetic test results will burden my family members	○	○	○	○	○
q. I feel that my genetic test results are too personal to share with my family members	○	○	○	○	○
r. I believe that it would be difficult for my family members to afford the cost of genetic testing for cancer risk	○	○	○	○	○
s. My family members have asked me not to talk with them about my genetic test results	○	○	○	○	○
t. I feel that nothing can be done to prevent cancer.	○	○	○	○	○

Spanish Version

Trial eligibility determination

¿Un médico o experto en genética le solicitó una prueba genética de sangre o saliva para detectar el riesgo de padecer cáncer?

☐ **Sí** Los resultados fueron **positivos** Tengo una mutación genética que aumenta el riesgo de cáncer	☐ **Sí** Los resultados fueron **inciertos** Se encontró una variante de significado incierto (VUS)	☐ **Sí** Los resultados fueron **negativos** No tengo mutaciones genéticas	☐ **No** Nunca me he hecho una prueba genética de cáncer

Family Members

¿Su **madre biológica** está viva en la actualidad y vive en Estados Unidos, Canadá o México?

☐ Sí

☐ No → Saltear a **C3** (Sus tías maternas – Hermanas de la madre)

Sus tías maternas (Hermanas de la madre)

A continuación, háblenos de sus **tías biológicas** de 18 años o más que vivan en el momento de la encuesta en Estados Unidos, Canadá o México.

Una tía materna es toda hermana biológica de su madre biológica. Esto no incluye a una tía que se haya casado con su familia.

C3 ¿Cuántas tiene?

☐ 0 ☐ 1 ☐ 2 ☐ 3 ☐ 4 ☐ 5 ☐ 6 ☐ 7 ☐ 8 ☐ 9 ☐ 10

Si tiene más de 10, escriba el número aquí: ☐☐

Sus tíos maternos (hermanos de la madre)

A continuación, háblenos de sus **tíos biológicos** de 18 años o más que vivan en el momento de la encuesta en Estados Unidos, Canadá o México.

Un tío materno es todo hermano biológico de su madre biológica. Esto no incluye a un tío que se haya casado con su familia.

C5 ¿Cuántos tiene?

☐ 0 ☐ 1 ☐ 2 ☐ 3 ☐ 4 ☐ 5 ☐ 6 ☐ 7 ☐ 8 ☐ 9 ☐ 10

Si tiene más de 10, escriba el número aquí: ☐☐

¿Su **padre biológico** está vivo en la actualidad y vive en Estados Unidos, Canadá o México?

☐ Sí

☐ No → Saltear a **D3** (Sus tías paternas – Hermanas del padre)

Sus tías paternas (Hermanas del padre)

A continuación, háblenos de sus **tías biológicas** de 18 años o más que vivan en el momento de la encuesta en Estados Unidos, Canadá o México.

> **Una tía paterna es toda hermana biológica de su padre biológico.** Esto no incluye a una tía que se haya casado con su familia.

D3 ¿Cuántas tiene?

☐ 0 ☐ 1 ☐ 2 ☐ 3 ☐ 4 ☐ 5 ☐ 6 ☐ 7 ☐ 8 ☐ 9 ☐ 10

Si tiene más de 10, escriba el número aquí: ☐☐

Sus tíos paternos (Hermanos del padre)

A continuación, háblenos de sus **tíos biológicos** de 18 años o más que vivan en el momento de la encuesta en Estados Unidos, Canadá o México.

> **Un tío paterno es todo hermano biológico de su padre biológico.** Esto no incluye a un tío que se haya casado con su familia.

D5 ¿Cuántos tiene?

☐ 0 ☐ 1 ☐ 2 ☐ 3 ☐ 4 ☐ 5 ☐ 6 ☐ 7 ☐ 8 ☐ 9 ☐ 10

Si tiene más de 10, escriba el número aquí: ☐☐

Sus hermanas

A continuación, háblenos de sus **hermanas biológicas** de 18 años o más que vivan en el momento de la encuesta en Estados Unidos, Canadá o México.

> **Una hermana biológica es una hermana con la misma madre o padre biológicos que usted. Esto incluye a las medio hermanas con las que solo comparte un progenitor.** No incluya a las hermanastras, hermanas adoptadas o cuñadas.

E1 ¿Cuántas tiene?

☐ 0 ☐ 1 ☐ 2 ☐ 3 ☐ 4 ☐ 5 ☐ 6 ☐ 7 ☐ 8 ☐ 9 ☐ 10

Si tiene más de 10, escriba el número aquí: ☐☐

Sus hermanos

A continuación, háblenos de sus **hermanos biológicos** de 18 años o más que vivan en el momento de la encuesta en Estados Unidos, Canadá o México.

> **Un hermano biológico es un hermano con la misma madre o padre biológicos que usted. Esto incluye a los medio hermanos con los que solo comparte un progenitor.** No incluya a los hermanastros, los hermanos adoptados o cuñados.

E3 ¿Cuántos tiene?

☐ 0 ☐ 1 ☐ 2 ☐ 3 ☐ 4 ☐ 5 ☐ 6 ☐ 7 ☐ 8 ☐ 9 ☐ 10

Si tiene más de 10, escriba el número aquí: ☐☐

Sus hijas

A continuación, háblenos de sus **hijas biológicas** de 18 años o más que vivan en el momento de la encuesta en Estados Unidos, Canadá o México.

> **Una hija biológica es una hija concebida por usted y nacida de usted.** No incluya hijastras, hijas adoptivas o nueras.

F1 ¿Cuántas tiene?

☐ 0 ☐ 1 ☐ 2 ☐ 3 ☐ 4 ☐ 5 ☐ 6 ☐ 7 ☐ 8 ☐ 9 ☐ 10

Si tiene más de 10, escriba el número aquí: ☐☐

Sus hijos

A continuación, háblenos de sus **hijos biológicos** de 18 años o más que vivan en el momento de la encuesta en Estados Unidos, Canadá o México.

> **Un hijo biológico es un hijo concebido por usted y nacido de usted.** No incluya a los hijastros, hijos adoptados o yernos.

F3 ¿Cuántos tiene?

☐ 0 ☐ 1 ☐ 2 ☐ 3 ☐ 4 ☐ 5 ☐ 6 ☐ 7 ☐ 8 ☐ 9 ☐ 10

Si tiene más de 10, escriba el número aquí: ☐☐

Sus sobrinas

A continuación, háblenos de sus **sobrinas biológicas** de 18 años o más que vivan en el momento de la encuesta en Estados Unidos, Canadá o México.

> **Una sobrina biológica es una hija biológica de su hermano o hermana.** No incluya a las sobrinas que no hayan sido concebidas por sus hermanos o hermanas ni hayan nacido de ellos, como sus hijas adoptadas o hijastras por matrimonio.

G1 ¿Cuántas tiene?

☐ 0 ☐ 1 ☐ 2 ☐ 3 ☐ 4 ☐ 5 ☐ 6 ☐ 7 ☐ 8 ☐ 9 ☐ 10

Si tiene más de 10, escriba el número aquí: ☐☐

Sus sobrinos

A continuación, háblenos de sus **sobrinos biológicos** de 18 años o más que vivan en el momento de la encuesta en Estados Unidos, Canadá o México.

> **Un sobrino biológico es un hijo biológico de su hermano o hermana.** No incluya a los sobrinos que no hayan sido concebidos por sus hermanos o hermanas ni hayan nacido de ellos, como sus hijos adoptivos o hijastros por matrimonio.

G3 ¿Cuántos tiene?

☐ 0 ☐ 1 ☐ 2 ☐ 3 ☐ 4 ☐ 5 ☐ 6 ☐ 7 ☐ 8 ☐ 9 ☐ 10

Si tiene más de 10, escriba el número aquí: ☐☐

SES Measures

¿Cuál de las siguientes opciones describe mejor su situación laboral **actual**?
Marque TODAS LAS OPCIONES que correspondan.

- ☐ Trabaja a tiempo completo (más de 40 horas a la semana)
- ☐ Trabaja a tiempo parcial (menos de 40 horas a la semana)
- ☐ Desempleado y buscando trabajo
- ☐ Despedido temporalmente o de baja por enfermedad u otro motivo
- ☐ Discapacitado
- ☐ Jubilado
- ☐ Estudiante
- ☐ Ama de casa

¿Qué tipo de seguro médico tiene actualmente? **Marque TODAS LAS OPCIONES que correspondan.**

- ☐ Ninguno
- ☐ Seguro brindado a través de mi empleador o sindicato actual o previo (incluida una Organización para el Mantenimiento de la Salud o HMO)
- ☐ Seguro brindado a otro integrante de la familia (por ejemplo, cónyuge) a través de su empleador o sindicato actual o previo (incluida una Organización para el Mantenimiento de la Salud o HMO)
- ☐ Seguro pagado directamente a una compañía de seguros (por usted u otro integrante de la familia)
- ☐ Seguro pagado a partir de un intercambio ("Obamacare" o la Ley del Cuidado de Salud a Bajo Precio)
- ☐ Medicaid u otro seguro brindado por el Estado (como Medi-Cal)
- ☐ Medicare/seguro del gobierno
- ☐ Indian Health Service
- ☐ TRICARE u otro servicio de atención médica militar
- ☐ Departamento de Asuntos de los Veteranos (VA, incluidos aquellos que han usado atención médica de VA o se inscribieron para recibirla)
- ☐ Otro (explique): ____________

Actualmente, ¿cuál es el ingreso total anual de su hogar, antes de descontar los impuestos, de todas las fuentes (incluidos los beneficios por manutención, pensión por alimentos, discapacidad, seguridad social y desempleo)?

- ☐ Menos de 5,000 USD
- ☐ Entre 5,000 y 9,999 USD
- ☐ Entre 10,000 y 19,999 USD
- ☐ Entre 20,000 y 29,999 USD
- ☐ Entre 30,000 y 39,999 USD
- ☐ Entre 40,000 y 59,999 USD
- ☐ Entre 60,000 y 89,999 USD
- ☐ Entre 90,000 y 119,999 USD
- ☐ 120,000 USD o más
- ☐ No lo sé

Actualmente, ¿cuántas personas viven a partir del ingreso total de su hogar, incluido usted mismo?

- ☐ 1 (solo usted)
- ☐ 2 personas
- ☐ 3 personas
- ☐ 4 o más personas

¿Cuál es el nivel educativo más alto que ha completado?

- ☐ Sin secundaria
- ☐ Secundaria incompleta
- ☐ Graduado de secundaria o GED
- ☐ Universidad o escuela técnica incompleta
- ☐ Graduado de la universidad (título de grado)
- ☐ Graduado de posgrado o superior

¿Qué sexo le asignaron al nacer, es decir, el que está en su acta original de nacimiento?

- ☐ Femenino
- ☐ Masculino
- ☐ Intersexual

¿Cuál de las siguientes opciones describe mejor su raza? **Marque TODAS LAS OPCIONES que correspondan.**

- ☐ Blanco
- ☐ Negro o afroamericano
- ☐ Indígena americano o nativo de Alaska
- ☐ Nativo de Hawaii o de otra isla del Pacífico
- ☐ Indoasiático
- ☐ Chino
- ☐ Filipino
- ☐ Japonés
- ☐ Coreano
- ☐ Vietnamita
- ☐ Otro asiático (explique): ______
- ☐ Otra etnia (explique): ______

¿Es usted de origen hispano, latino o español?

- ☐ Sí, mexicano, mexicoestadounidense o chicano
- ☐ Sí, puertorriqueño
- ☐ Sí, cubano
- ☐ Sí, otro tipo de hispano, latino o español (explique): ______
- ☐ No

Assessment of Family Communication Scale

Si piensa en hablar con su familia biológica sobre las pruebas genéticas de riesgo de cáncer, ¿en qué medida son ciertas las siguientes afirmaciones?

(Marque con una X una casilla de cada fila)

	No es cierto	Un poco cierto	Algo cierto	Bastante cierto	Muy cierto
a. Entiendo los resultados de mis pruebas genéticas lo suficientemente bien como para hablar de ellos con mis familiares	☐	☐	☐	☐	☐
b. Me siento cómodo hablando con mis familiares sobre los resultados de mis pruebas genéticas	☐	☐	☐	☐	☐
c. Confío en poder hablar con mis familiares sobre las pruebas genéticas	☐	☐	☐	☐	☐
d. Tengo tiempo suficiente para acercarme a mis familiares y hablar con ellos sobre las pruebas genéticas	☐	☐	☐	☐	☐
e. Mi familia se siente cómoda hablando de las pruebas genéticas de riesgo de cáncer	☐	☐	☐	☐	☐
f. Puedo manejar las emociones de los miembros de mi familia al hablar de las pruebas genéticas	☐	☐	☐	☐	☐
g. Puedo manejar mis propias emociones cuando hablo con mis familiares sobre las pruebas genéticas	☐	☐	☐	☐	☐
h. No sé con qué miembros de la familia debo hablar sobre las pruebas genéticas de riesgo de cáncer	☐	☐	☐	☐	☐
i. Siento que no puedo hacer nada más para animar a los miembros de mi familia a hacerse las pruebas genéticas	☐	☐	☐	☐	☐
j. Mis familiares me han preguntado por los resultados de mis pruebas genéticas	☐	☐	☐	☐	☐
k. Creo que las pruebas genéticas pueden ayudar a prevenir el cáncer en los miembros de mi familia	☐	☐	☐	☐	☐
l. Quiero que los miembros de mi familia se sometan a pruebas genéticas	☐	☐	☐	☐	☐
m. Un profesional de la salud o consejero genético me animó a compartir los resultados de mis pruebas genéticas con mis familiares	☐	☐	☐	☐	☐
n. Creo que los resultados de mis pruebas genéticas son útiles para mis familiares	☐	☐	☐	☐	☐
o. Considero que es mi responsabilidad compartir los resultados de mis pruebas genéticas con los miembros de mi familia	☐	☐	☐	☐	☐
p. Creo que compartir los resultados de mis pruebas genéticas será una carga para los miembros de mi familia	☐	☐	☐	☐	☐
q. Siento que los resultados de mis pruebas genéticas son demasiado personales para compartirlos con mis familiares	☐	☐	☐	☐	☐
r. Creo que a los miembros de mi familia les resultará difícil costear las pruebas genéticas de riesgo de cáncer	☐	☐	☐	☐	☐
s. Mis familiares me han pedido que no hable con ellos sobre los resultados de mis pruebas genéticas	☐	☐	☐	☐	☐
t. Creo que no se puede hacer nada para prevenir el cáncer.	☐	☐	☐	☐	☐

Appendix C: Relative Eligibility Screener

Note: Items created de novo for this study

English Version

1. We want to confirm our GIFT email was delivered to the right person. Are you [relative first name and last name]?

- Yes
- No *(screens out)*

2. Please indicate your relationship to [the enrolled patient who invited this relative]. *I am this person's:*

- Mother
- Father
- Sister
- Half-sister (we only share 1 parent)
- Brother
- Half-brother (we only share 1 parent)
- Daughter
- Son
- Aunt
- Uncle
- Niece
- Nephew
- Grandmother
- Granddaughter
- Grandson
- Other/None of the above *(screens out)*

3. What is your current age?

- 17 years or younger *(screens out)*
- 18-24
- 25-29
- 30-39
- 40-49
- 50-59
- 60-64
- 65-69
- 70-79
- 80-89
- 90-99
- 100+

4. In what country do you currently live?

- United States (including Puerto Rico and other territories)
- Canada (including territories)
- Mexico
- Another country *(screens out)*

5. Genetic testing for cancer risk is ordered by a doctor or genetic expert to look for inherited gene changes that might put a person at higher risk of getting certain kinds of cancer. These gene changes are called "pathogenic variants" or "genetic mutations". The test is ordered by a doctor or genetics expert and is done on a saliva (spit) or blood sample.

In the past 5 years, have you had a blood or saliva (spit) genetic test for cancer risk that was **ordered by a doctor or genetics expert?**

- Yes *(screens out)*
- No
- Don't know

Spanish Version

¡Gracias por demostrar interés en ser parte del estudio GIFT! Para comenzar, le haremos algunas preguntas para asegurarnos de que el estudio GIFT sea adecuado para usted.
1. Queremos confirmar que nuestro correo de GIFT se haya enviado a la persona correcta. ¿Usted es _____?
- Sí
- No

2. Indique su relación con ________. *Soy .... de esta persona:*
- Madre
- Padre
- Hermana
- Medio hermana (solo compartimos 1 progenitor)
- Hermano
- Medio hermano (solo compartimos 1 progenitor)
- Hija
- Hijo
- Tía
- Tío
- Sobrina
- Sobrino
- Abuela
- Nieta
- Nieto
- Otro/ninguno de los anteriores

3. ¿Cuántos años tiene?
- 17 años o menos
- Entre 18 y 24
- Entre 25 y 29
- Entre 30 y 39
- Entre 40 y 49
- Entre 50 y 59
- Entre 60 y 64
- Entre 65 y 69
- Entre 70 y 79
- Entre 80 y 89
- Entre 90 y 99
- Más de 100

4. ¿En qué país vive?
- Estados Unidos (incluidos Puerto Rico y otros territorios)
- Canadá (incluidos los territorios)
- México
- Otro país

5. Un médico o un experto en genética solicita la prueba genética para detectar el riesgo de padecer cáncer para detectar si hay cambios genéticos heredados que puedan poner a una persona en mayor riesgo de padecer ciertos tipos de cáncer. Estos cambios genéticos se llaman “variantes patógenas” o “mutaciones genéticas”. Un médico o experto en genética solicita esta prueba y se realiza con una muestra de saliva o sangre.

En los últimos 5 años, ¿un médico o experto en genética le solicitó una prueba genética de sangre o saliva para detectar el riesgo de padecer cáncer?

- Sí
- No
- No lo sé

Appendix D: Relative Baseline Measures

Note: Items created de novo for this study

English Version

1. **Before you were invited to join the GIFT Study, did you…**

O Yes O No a. Know about [the patient who invited you into the study]'s history of cancer?

O Yes O No b. Know that [the patient who invited you into the study] had been found to carry a gene change or mutation that increases the risk of cancer?

2. **Before you were invited to join the GIFT Study,** how much did you understand that [the patient who invited you into the study's] genetic test results mean that you *could* have an increased risk of cancer *if you have the same genetic mutation*?

Did not understand this at all	Understood a little bit	Understood somewhat	Understood quite a bit	Understood very much

4. **Before you were invited to join the GIFT Study**, how much had you considered getting a genetic test for cancer risk?

Not at all	A little bit	Somewhat	Quite a bit	Very much

5. **As of right now**, how much do you want to get a genetic test for cancer risk?

Not at all	A little bit	Somewhat	Quite a bit	Very much

Spanish Version

1. **Antes de que lo invitaran a ser parte del estudio GIFT, usted...**

O Sí O No a. ¿Sabía de los antecedentes de cáncer de [probando]?

O Sí O No b. ¿Sabía que se detectó que [probando] tiene un cambio o mutación genética que aumenta el riesgo de padecer cáncer?

2. **Antes de que lo invitaran a ser parte del estudio GIFT,** ¿qué tanto comprendía que los resultados de la prueba genética de [probando] implicaban que usted *podía* tener un mayor riesgo de padecer cáncer *si tenía la misma mutación genética*?

No lo entendía en absoluto	Lo entendía muy poco	Lo entendía un poco	Lo entendía bastante	Lo entendía muy bien

4. **Antes de que lo invitaran a ser parte del estudio GIFT,** ¿qué tanto pensó en realizarse una prueba genética para verificar su riesgo de padecer cáncer?

Nada	Muy poco	Un poco	Bastante	Mucho

5. **Ahora mismo,** ¿qué tanto quiere realizarse una prueba genética para verificar el riesgo de padecer cáncer?

Nada	Muy poco	Un poco	Bastante	Mucho

Appendix E: Patient Follow-Up Survey Measures

English Version

Assessment of Family Communication Scale

Scale created de novo for this study

I2. Thinking about talking with your biological family about genetic testing for cancer risk, how true are each of the statements below?

	Not at all true	A little true	Somewhat true	Quite true	Very true
a. I understand my genetic test results well enough to talk about them with my family members	○	○	○	○	○
b. I am comfortable talking to my family members about my genetic test results	○	○	○	○	○
c. I am confident that I can talk with my family members about genetic testing	○	○	○	○	○
d. I have enough time to reach out to my family members and talk with them about genetic testing	○	○	○	○	○
e. My family is comfortable talking about genetic testing for cancer risk	○	○	○	○	○
f. I can handle the emotions of my family members when talking about genetic testing	○	○	○	○	○
g. I can handle my own emotions when talking with my family members about genetic testing	○	○	○	○	○
h. I don't know which family members I should talk with about genetic testing for cancer risk	○	○	○	○	○
i. I feel as though there's nothing more I can do to encourage my family members to get genetic testing	○	○	○	○	○
j. My family members have asked me about my genetic test results	○	○	○	○	○
k. I believe that genetic testing can help prevent cancer in my family members	○	○	○	○	○
l. I want my family members to get genetic testing	○	○	○	○	○
m. A health care provider or genetic counselor encouraged me to share my genetic test results with my family members	○	○	○	○	○
n. I believe that my genetic test results are useful to my family members	○	○	○	○	○

o. I feel that it is my responsibility to share my genetic test results with my family members	○	○	○	○	○
p. I believe that sharing my genetic test results will burden my family members	○	○	○	○	○
q. I feel that my genetic test results are too personal to share with my family members	○	○	○	○	○
r. I believe that it would be difficult for my family members to afford the cost of genetic testing for cancer risk	○	○	○	○	○
s. My family members have asked me not to talk with them about my genetic test results	○	○	○	○	○
t. I feel that nothing can be done to prevent cancer.	○	○	○	○	○

Spanish Version

Assessment of Family Communication Scale

Si piensa en hablar con su familia biológica sobre las pruebas genéticas de riesgo de cáncer, ¿en qué medida son ciertas las siguientes afirmaciones?

	(Marque con una X una casilla de cada fila)				
	No es cierto	Un poco cierto	Algo cierto	Bastante cierto	Muy cierto
a. Entiendo los resultados de mis pruebas genéticas lo suficientemente bien como para hablar de ellos con mis familiares	☐	☐	☐	☐	☐
b. Me siento cómodo hablando con mis familiares sobre los resultados de mis pruebas genéticas	☐	☐	☐	☐	☐
c. Confío en poder hablar con mis familiares sobre las pruebas genéticas	☐	☐	☐	☐	☐
d. Tengo tiempo suficiente para acercarme a mis familiares y hablar con ellos sobre las pruebas genéticas	☐	☐	☐	☐	☐
e. Mi familia se siente cómoda hablando de las pruebas genéticas de riesgo de cáncer	☐	☐	☐	☐	☐
f. Puedo manejar las emociones de los miembros de mi familia al hablar de las pruebas genéticas	☐	☐	☐	☐	☐
g. Puedo manejar mis propias emociones cuando hablo con mis familiares sobre las pruebas genéticas	☐	☐	☐	☐	☐
h. No sé con qué miembros de la familia debo hablar sobre las pruebas genéticas de riesgo de cáncer	☐	☐	☐	☐	☐

i. Siento que no puedo hacer nada más para animar a los miembros de mi familia a hacerse las pruebas genéticas	☐	☐	☐	☐	☐
j. Mis familiares me han preguntado por los resultados de mis pruebas genéticas	☐	☐	☐	☐	☐
k. Creo que las pruebas genéticas pueden ayudar a prevenir el cáncer en los miembros de mi familia	☐	☐	☐	☐	☐
l. Quiero que los miembros de mi familia se sometan a pruebas genéticas	☐	☐	☐	☐	☐
m. Un profesional de la salud o consejero genético me animó a compartir los resultados de mis pruebas genéticas con mis familiares	☐	☐	☐	☐	☐
n. Creo que los resultados de mis pruebas genéticas son útiles para mis familiares	☐	☐	☐	☐	☐
o. Considero que es mi responsabilidad compartir los resultados de mis pruebas genéticas con los miembros de mi familia	☐	☐	☐	☐	☐
p. Creo que compartir los resultados de mis pruebas genéticas será una carga para los miembros de mi familia	☐	☐	☐	☐	☐
q. Siento que los resultados de mis pruebas genéticas son demasiado personales para compartirlos con mis familiares	☐	☐	☐	☐	☐
r. Creo que a los miembros de mi familia les resultará difícil costear las pruebas genéticas de riesgo de cáncer	☐	☐	☐	☐	☐
s. Mis familiares me han pedido que no hable con ellos sobre los resultados de mis pruebas genéticas	☐	☐	☐	☐	☐
t. Creo que no se puede hacer nada para prevenir el cáncer.	☐	☐	☐	☐	☐

Appendix F: Relative Follow-Up Survey Measures

English Version

- In the past six months, have you had a counseling visit with a genetics expert to discuss cancer risk – that is, an appointment where the whole discussion is about genetic risk for cancer?

O	O
Yes	No

Katz SJ, Ward KC, Hamilton AS, Mcleod MC, Wallner LP, Morrow M, Jagsi R, Hawley ST, Kurian AW. Gaps in Receipt of Clinically Indicated Genetic Counseling After Diagnosis of Breast Cancer. J Clin Oncol. 2018 Apr 20;36(12):1218-1224. doi: 10.1200/JCO.2017.76.2369. Epub 2018 Mar 12. PMID: 29528794; PMCID: PMC5908222.

Spanish Version

En los últimos seis meses, ¿ha consultó a un experto en genética para hablar del riesgo de padecer cáncer? (Es decir, una cita donde toda la conversación se tratara del riesgo genético de padecer cáncer).

O Sí O No
